# Photoreceptor cKO of OTX2 Enhances OTX2 Intercellular Transfer in the Retina and Causes Photophobia

**DOI:** 10.1523/ENEURO.0229-21.2021

**Published:** 2021-10-06

**Authors:** Pasquale Pensieri, Annabelle Mantilleri, Damien Plassard, Takahisa Furukawa, Kenneth L. Moya, Alain Prochiantz, Thomas Lamonerie

**Affiliations:** 1 Université Côte d’Azur, Centre National de la Recherche Scientifique, Institut National de la Santé et de la Recherche Médicale, Institut de Biologie Valrose, Nice 06108, France; 2 Plateforme GenomEast, Institut de Génétique et de Biologie Moléculaire et Cellulaire, Illkirch 67404, France; 3 Laboratory for Molecular and Developmental Biology, Institute for Protein Research, Osaka University, Osaka 565-0871, Japan; 4 Centre for Interdisciplinary Research in Biology (CIRB), Collège de France, Centre National de la Recherche Scientifique, Unité Mixte de Recherche 7241, Institut National de la Santé et de la Recherche Médicale Unité 1050, Paris 75005, France

**Keywords:** arrestin-1, homeoprotein transfer, mouse, Otx2, photoreceptors, retina

## Abstract

In the mature mouse retina, *Otx2* is expressed in both retinal pigmented epithelium (RPE) and photoreceptor (PR) cells, and *Otx2* knock-out (KO) in the RPE alone results in PR degeneration. To study the cell-autonomous function of OTX2 in PRs, we performed PR-specific *Otx2* KO (cKO) in adults. As expected, the protein disappears completely from PR nuclei but is still observed in PR inner and outer segments while its level concomitantly decreases in the RPE, suggesting a transfer of OTX2 from RPE to PRs in response to *Otx2* ablation in PRs. The ability of OTX2 to transfer from RPE to PRs was verified by viral expression of tagged-OTX2 in the RPE. Transferred OTX2 distributed across the PR cytoplasm, suggesting functions distinct from nuclear transcription regulation. PR-specific *Otx2* cKO did not alter the structure of the retina but impaired the translocation of PR arrestin-1 on illumination changes, making mice photophobic. RNA-seq analyses following *Otx2* KO revealed downregulation of genes involved in the cytoskeleton that might account for the arrestin-1 translocation defect, and of genes involved in extracellular matrix (ECM) and signaling factors that may participate in the enhanced transfer of OTX2. Interestingly, several RPE-specific OTX2 target genes involved in melanogenesis were downregulated, lending weight to a decrease of OTX2 levels in the RPE following PR-specific *Otx2* cKO. Our study reveals a new role of endogenous OTX2 in PR light adaptation and demonstrates the existence of OTX2 transfer from RPE to PR cells, which is increased on PR-specific *Otx2* ablation and might participate in PR neuroprotection.

## Significance Statement

OTX2 homeoprotein is expressed in retinal pigmented epithelium (RPE), photoreceptors (PRs) and bipolar cells (BCs). The function of endogenous PR OTX2, which, in contrast with RPE OTX2, is dispensable for PR maintenance, is unknown. We performed PR-specific *Otx2* knock-out (KO) and found that removal of endogenous PR OTX2 leads to impaired arrestin-1 translocation associated with photophobia, specific modifications of PR and RPE gene expression, and to increased transfer of OTX2 protein from the RPE to the PR cytoplasm. Thus, several PR activities, including light adaptation, rely on endogenous nuclear OTX2, while PR neuroprotection seems to require RPE OTX2, highlighting the importance of non-cell-autonomous OTX2 in the adult retina.

## Introduction

OTX2 belongs to the bicoid family of homeoproteins, a large group of evolutionarily ancient proteins characterized by the homeodomain, a 60-amino acid DNA-binding domain, that primarily act as transcription factors (Di Nardo et al., 2018). OTX2 plays essential roles throughout brain and retinal development (Béby and Lamonerie, 2013). In the mammalian retina, *Otx2* expression is critical for retinal pigmented epithelium (RPE) specification, photoreceptor (PR) differentiation and bipolar cell (BC) maturation (Martinez-Morales et al., 2001; Nishida et al., 2003; Koike et al., 2007). In the adult retina, *Otx2* is expressed in the RPE, in most BCs and in all cone and rod PRs (Fossat et al., 2007), where it exerts cell-autonomous and non-cell-autonomous activities (Housset et al., 2013; Kim et al., 2015; Torero Ibad et al., 2020). In particular, *Otx2* expression in the RPE is critical for long-term survival of PRs, which otherwise slowly degenerate in its absence (Béby et al., 2010). RPE-specific *Otx2* KO and RPE-specific expression of *Otx2* in full KO retinas demonstrated that RPE OTX2 is necessary and sufficient for PR maintenance, while its expression in PRs (and BCs) appears dispensable for their survival (Housset et al., 2013). In the RPE, OTX2 coordinates the expression of genes controlling melanogenesis, visual cycle, metal homeostasis and pH regulation, many of them associated with human retinal diseases (Housset et al., 2013). Given the importance of the maintenance functions of the RPE (Strauss, 2005), cell-autonomous defects of the RPE in the absence of OTX2, could well explain the PR death. However, this does not rule out a non-autonomous action of OTX2 produced by the RPE, nor does it shed any light on the role of OTX2 produced by PRs.

Many homeoproteins, including OTX2, can be transferred to cells that do or do not express them (Lee et al., 2019). Several studies have highlighted the importance of exogenous OTX2 for controlling the plasticity of the visual cortex, the maturation of specific BC and the neuroprotection of retinal ganglion cells (RGCs; Sugiyama et al., 2008; Torero Ibad et al., 2011; Kim et al., 2015). Exogenous OTX2 has been shown to work as a canonical transcription factor in target cells (Apulei et al., 2019) but also as a non-canonical extranuclear factor, for instance in mitochondrial energy complex stabilization (Kim et al., 2015). A proteomic study showing OTX2 association with proteins of the mitochondrial energy production complexes and the neurotransmitter secretion machinery in the retina adds support to non-genomic and extracellular functions of this factor (Fant et al., 2015). Whether OTX2 transfer from RPE to PR cells occurs and participates in their neuroprotection is not known.

PRs are specialized neurons that absorb photons and convert light into visual information conveyed to the brain via BCs and RGCs. They develop outer segments that are in contact with the RPE through a specialized extracellular matrix (ECM), the inter-PR matrix (IPM; Ishikawa et al., 2015). PR outer segments are filled with membranous discs that support the proteins involved in phototransduction, including opsin, transducin, rhodopsin kinase, and arrestin. Some of these proteins can translocate back and forth between the outer segment and inner compartments, depending on illumination conditions, to achieve maximal light sensitivity and protection against phototoxicity (Gurevich et al., 2011). PRs are highly energetic cells and their survival relies on proper supply of nutrients and energy production (Country, 2017). *Otx2* expression is maintained in PRs throughout life, raising the question of its intrinsic function. We addressed this by specifically removing OTX2 from mature PR cells and show that this operation affects the translocation of arrestin-1 in response to illumination changes, causing photophobia, and indirectly impacts RPE cells by increasing the transfer of OTX2 from the RPE to inner retinal regions. Together, our results provide evidence of both intrinsic and extrinsic roles for OTX2 in adult PRs.

## Materials and Methods

### Animals

All mouse strains were maintained on a 129/SV background and housed in a 12/12 h light/dark cycle. PR-specific *Otx2* cKO mice were generated by breeding *Crx*-*CreERT2* transgenic mice (Muranishi et al., 2011) obtained from the Riken with *Otx2*^flox/flox^ mice (Fossat et al., 2006). CreERT2 activation was induced at P30 or at the indicated age by a single intraperitoneal injection of tamoxifen (Sigma-Aldrich; 10 mg/ml in sunflower oil) at 100 μg/g of body weight. The *R26^Ai14^* Cre-reporter line *Gt(ROSA)^26Sortm14(CAG-tdTomato)Hze^/*J (Madisen et al., 2010) was purchased from JAX. For all experiments, mice of both sexes were used without distinction. Animal care and experiments were conducted under the protocol PEA#203, approved by the French Government and local ethics committee CIEPAL-Azur, in accordance with the European Communities Council Directive of 22 September 2010 (2010/63/EEC).

### Immunocytochemistry and histologic studies

Eyes were collected and placed in cold PBS, punctured at the level of the ora serrata, fixed in 4% paraformaldehyde (PFA) in PBS for 2 h at room temperature (RT), rinsed three times in PBS, cryo‐protected overnight at 4°C in PBS-sucrose 20%, frozen in Tissue-Tek OCT and stored at –80°C. Sections (14 μm) were mounted onto SuperFrost+ slides (Fisher Scientific). Slides were dried for 1 h at RT, washed three times 5 min in PBS with 1% Triton X-100 (PBST), placed in PBS-100 mm glycine for 30 min at RT, blocked 1 h in PBST containing 10% fetal bovine serum (FBS), and incubated overnight at 4°C with the primary antibodies diluted in PBST with 10% FBS. The next day, slides were washed three times 10 min in PBST at RT, incubated 2 h at RT with the secondary antibodies diluted in PBST with 10% FBS and processed with DAPI staining (5 min at RT in solution of 1 μg/ml in PBS), before mounting in Fluoromount-G (ThermoFisher Scientific). Images were acquired using a wide-field Zeiss AxioPlan microscope or laser scanning confocal microscopes Leica SP5 and Zeiss LSM780.

#### Primary antibodies

OTX2 (goat, 1:500): R&D Systems AF1979, arrestin-1 (mouse, 1:1000): Santa Cruz sc-166383, Rhodopsin (rabbit, 1:1000): Genetex GTX129910, M-Opsin (rabbit, 1:1000): Abcam AB5405, S-Opsin (rabbit, 1:1000): Abcam AB5407, Chx10 (mouse, 1:250): Santa Cruz sc-365519, Crx (rabbit, 1:250): Sigma HPA036762, DS-Red (rabbit, 1:500): Takara (OZYME) 632496, Brn3A (goat, 1:250): Abcam AB144, Pax6 (rabbit, 1:500) Millipore, AB2237, Calbindin (mouse, 1:500): Sigma C9848, and GFAP (rabbit, 1:1000): Invitrogen PA1-10 019.

#### Secondary antibodies

Donkey anti-rabbit (Cy3) 1:500 Jackson ImmunoResearch (JI) 711-065-152, donkey anti-rabbit (Cy5) 1:500 JI 711-065-152, donkey anti-rabbit (Alexa Fluor 488) 1:500 JI 711-065-152, donkey anti-goat (Cy3) 1:500 JI 705-165-147, and donkey anti-mouse (Cy3) 1:500 JI 715-165-150.

For histologic studies, eyes were processed as above. Hematoxylin/eosin staining was performed according to standard protocol. Images were acquired on the AxioPlan microscope (Zeiss) with a color camera.

### Light-dark adaptation

For dark adaptation, mice were exposed to diffuse white light (900-lux intensity at the level of cage lid) for at least 8 h, then put in the dark. Mice were killed and eyes were collected under dim red light at indicated times after dark transition. For light adaptation, mice were put in the dark for at least 8 h, then exposed to diffuse white light (900-lux intensity at the level of cage lid) for the indicated times before eye collection.

### Behavioral tests

Testing was conducted using 7- to 10-week-old littermates that had received one intraperitoneal injection of tamoxifen at P30 (Sigma-Aldrich; 10 mg/ml in sunflower oil, 100 μg/g of body weight). Animals were acclimated for 30 min to the behavioral room before all experiments.

#### Light-dark box test

Dark-adapted mice were individually tested for a total of 25 min in a light/dark box. The box was composed of two compartments: one brightly lit (150 lux) of 19.5-cm width (W) × 29.5-cm length (L) × 30-cm height (H), painted white and lacking a top, the other not lit of 19.5-cm W × 14.5-cm L × 30-cm H, painted black and fully enclosed. A small opening (5 × 5 cm) connecting the two compartments allowed mice to change compartments freely. Before testing, mice were acclimated 30 min in their cages in the testing room in the dark. Each mouse was gently placed in the center of the open side of the box, facing away from the dark side. Mice were video tracked for a total time of 25 min by EthoVision software. The first 10 min allowed mice to explore compartments. An object (white plastic cylinder of 5 cm in diameter, 4 cm high) was then placed in the right corner of the light compartment, opposite to the dark compartment for 5 min. The object was then moved to the right corner of the dark compartment. The object was removed for the last 5 min. The video tracking system was used to monitor behavior and quantify the time spent in the light versus the dark compartment for the entire duration of the test.

#### Openfield test

Mice adapted to normal light conditions were tested for 10 min in an empty and bright square arena (40-cm W × 40-cm L × 30-cm H), surrounded by black walls. A light placed above the open field gave a 100-lux luminance evenly distributed in the open field. Animals were individually placed in the center of the arena and video tracked for 10 min by EthoVision software. Time spent in the center and in the periphery (set as 5 cm from walls) as well as distances traveled and velocity were measured. Anxiety-related behavior was measured by the degree to which the mouse avoided the center area.

### Expression analyses

#### RNA preparation

Control or tamoxifen-injected mice (*n* = 3 for each condition) were killed 0, 2, 4, and 8 d after tamoxifen injection at 3 P.M. Eyes were collected, dissected in cold PBS and fresh retinas were placed in TRI reagent (Sigma). Total RNA was extracted according to manufacturer’s protocol.

#### Semi-quantitative RT-PCR

First-strand cDNA was synthesized using 1 μg of RNA, 5 units of MLV reverse transcriptase (Promega) and 100 ng of random hexamers. For each PCR, 20 ng cDNA were used with Taq Polymerase and specific genes, oligonucleotides in the following PCR conditions: initial denaturation 94°C for 3 min, 30 PCR cycles (15 s at 94°C, 30 s at 60°C, 30 s at 72°C) with an extension step at the end of the 30 cycles (5 min at 72°C). Reactions were run on 1.5% agarose gels stained with ethidium bromide and acquired on a UV imager.

#### PCR oligonucleotides

The following primers were used: *Tyrosinase*, 5′-ATTGATTTTGCCCATGAAGCA-3′ (forward) and 5′-TTCCATCGCATAAAACCTGAT-3′ (reverse); *Tyrp-1*, 5′-TTCACTGATGCGGTCTTTGA-3′ (forward) and 5′-CGAAAATGGCAGCTACAAGT-3′ (reverse); *Mlana*, 5′-ACTGCTGAAGAGGCCGCAGG-3′ (forward) and 5′-TTGGGAACCACGGGCTGATG-3′ (reverse); and *HGPRT*, 5′-ATGAGTACTTCAGGGATTTGA-3′ and 5′-TAAGCGACAATCTACCAGAG-3′ (reverse).

#### RNA sequencing and data analyses

Total retinal RNA was prepared in triplicate from three mice for each condition. Libraries were prepared from 500 ng of total RNA using TruSeq Stranded mRNA LT Sample Preparation kit (Illumina), according to manufacturer’s instructions. Final cDNA libraries were checked for quality and quantified using capillary electrophoresis. Single-end sequencing (50 bp) was performed on the GenomEast Illumina HiSeq 4000 platform. Image analysis and base calling were performed using RTA 2.7.3 and bcl2fastq 2.17.1.14 For alignment, reads were mapped onto mm10 assembly of the mouse genome using STAR v.2.5.3a. HTSeq v.0.6.1p1 software (union mode) and Ensembl 93 release version were used for gene expression quantification and annotation. Comparisons of normalized read counts were performed using R 3.3.2 with DESeq2 v1.16.1 Bioconductor package. Principal Component Analysis was computed on regularized logarithm transformed data calculated with the DESeq2 method. To estimate efficiency of the deletion, for each sample, read numbers for the floxed exon E5 and for the closest neighboring exon E4 were extracted. To compare different samples, the E5/E4 reads ratio of each sample was calculated. For each time point, the mean E5/E4 reads ratio and SD of the triplicate were determined. The % reduction of this ratio at P32, P34, and P38 was obtained by comparing to the E5/E4 reads ratio at P30. RNA-seq data are deposited on GEO (access number GSE 138097). Only genes with a *p* ≤ 0.05 were considered to be changed. All genes with a log2FC ≤ −0.5 were considered downregulated and genes with a log2FC ≥ 0.5 were considered upregulated. The list of ranked genes was processed for gene enrichment and pathway analyses using Gene Ontology (www.geneontology.org), ImPALA (impala.molgen.mpg.de), and GSEA (https://www.gsea-msigdb.org/gsea/index.jsp) softwares. ClueGO (https://apps.cytoscape.org/apps/cluego) was used to group genes into functional classes. The Heatmaps were established using DEseq2 normalized adjusted read counts with Phantasus (https://artyomovlab.wustl.edu/phantasus/).

### AAV vectors

The soluble alkaline phosphatase tag (SoAP) was created by removing the seven leucine residues at the N terminus of SEAP by PCR mutagenesis on pSEAP-basic vector (Takara Bio) with primers designed to add Not I sites at both ends. *OTX2-SoAP* was obtained by fusing the PCR-amplified OTX2 coding region, without its stop codon, in frame with PCR-amplified SoAP coding sequence. SoAP and OTX2-SoAP fusion coding sequences were substituted to GFP in pTR-*Vmd2-GFP* (a generous gift of Dr. W. Hauswirth) to obtain the AAV2-*Vmd2-SoAP* and *AAV2-Vmd2-OTX2-SoAP* donor vectors. All primer information is available on request.

#### AAV production

Donor plasmids were transiently transfected into 293AAV cells together with AAV-Helper and AAV-DJ REP-CAP plasmids (Cell Biolabs). Recombinant AAVs were purified and titrated according to the manufacturer instructions. For each vector, two 10-cm plates were transfected and used for particle production. The average concentration obtained was 5 × 10^8^ genome copies/μl.

### Subretinal injections

Purified AAV vectors were administrated by subretinal injection in the right eye. The left eye was kept as internal negative control. Eight-week-old mice were anesthetized by intraperitoneal injection of a mixture of Rompun 2% (5 μl/g) and buprenorphine (0.1 mg/kg) for analgesia. One drop of tetracaine 1% (unidose collyre, Virbac) was put on each eye for local anesthesia. The pupils were dilated using a drop of neosynephrine 10% (Faure) and a drop of atropine 1% (VT dose, Virbac). The cornea was covered with a drop of Ocry-gel (Tvm lab) and a glass cover slip. Under a binocular microscope, the right eye was punctured at the corneal–scleral junction with a 5-μl Hamilton syringe mounted with a beveled 34-gauge needle. The needle was inserted into the subretinal space to reach its posterior part, at the opposite of the hole. A total of 2 μl of AAV suspension was delivered to create a subretinal bleb, and the needle was left in place 30 s before withdrawal. The eyes were injected with AAV_DJ_-*Vmd2-OTX2-SOAP* or AAV_DJ_-*Vmd2- SOAP* as control at 1.35 × 10^7^ GC/μl.

### Alkaline phophatase detection and quantification

Eyes transduced with AAV particles were processed as above. Frozen slides were dried 1 h at RT and washed three times 5 min in PBS. Endogenous phosphatases were inactivated by 20-min incubation at 65°C. To ensure a better contrast of the NBT/BCIP staining in the RPE layer, pigment was bleached by incubating the slides 10 min at RT in bleaching solution (5% formamide, 0.5× SSC, and 7.5% H_2_O_2_). Slides were then washed three times 5 min in PBS, equilibrated 1 h in alkaline NTMT solution (100 mm NaCl, 100 mm Tris, pH 9.5, 1% Tween 20, and 50 mm MgCl_2_) and incubated with AP‐substrates NBT/BCIP (Roche) 20 min at 37°C. Slides were washed 2 min in H_2_O and mounted in Mowiol. Pictures were acquired on the AxioPlan microscope (Zeiss) with a color camera.

### Imaging, experimental design, and statistical analysis

All series of images used for comparison were processed for immunohistochemistry simultaneously and acquired with the same settings. Images were captured on a laser scanning confocal microscope (Leica SP5) or on a Wide field upright microscope coupled to a color camera (Zeiss axioplan2). Confocal images are maximal intensity projections made by taking the greatest pixel intensity of each xyz point of a 3D image created from the Z-stack and combining them into a 2D image.

For quantifications, images were processed using ImageJ software as explained in the figure legends. The size of samples (*n*) in each experiment presented is specified in each figure legend.

For each animal, three sections were used for quantification and on each section, a minimum of three normalized fields (50 μm wide encompassing the RPE and PR layers) in the region of Interest were processed using ImageJ. For OTX2 quantification, the region of interest was the whole section, for SoAP quantification, the region of interest was chosen within the central 80% of the width of the transduced region avoiding the 10% on each side. Background signal measured on a similar non-stained area of the same section was subtracted. Then the signal in a given area was determined and expressed as its proportion to the total normalized signal of the field. All quantifications are presented as the mean. Error bars indicate SD. Before using Student’s *t* test to compare two groups of data, such as mutants and controls, unequal variance was first assessed by performing the *F* test. All statistical comparisons rejected unequal variance. Unpaired, two-tailed *t* test assuming equal variance was then used to compare mean data from two groups. Minimal statistical significance was fixed at *p* < 0.05; *p* values are represented on the graphs as follows: *p < 0.05, **p < 0.01, ***p < 0.001. All statistical analyses are presented in Table 3.

## Results

### *Otx2* deletion in PRs reveals OTX2 protein transfer from RPE to PR

**Figure 1.**
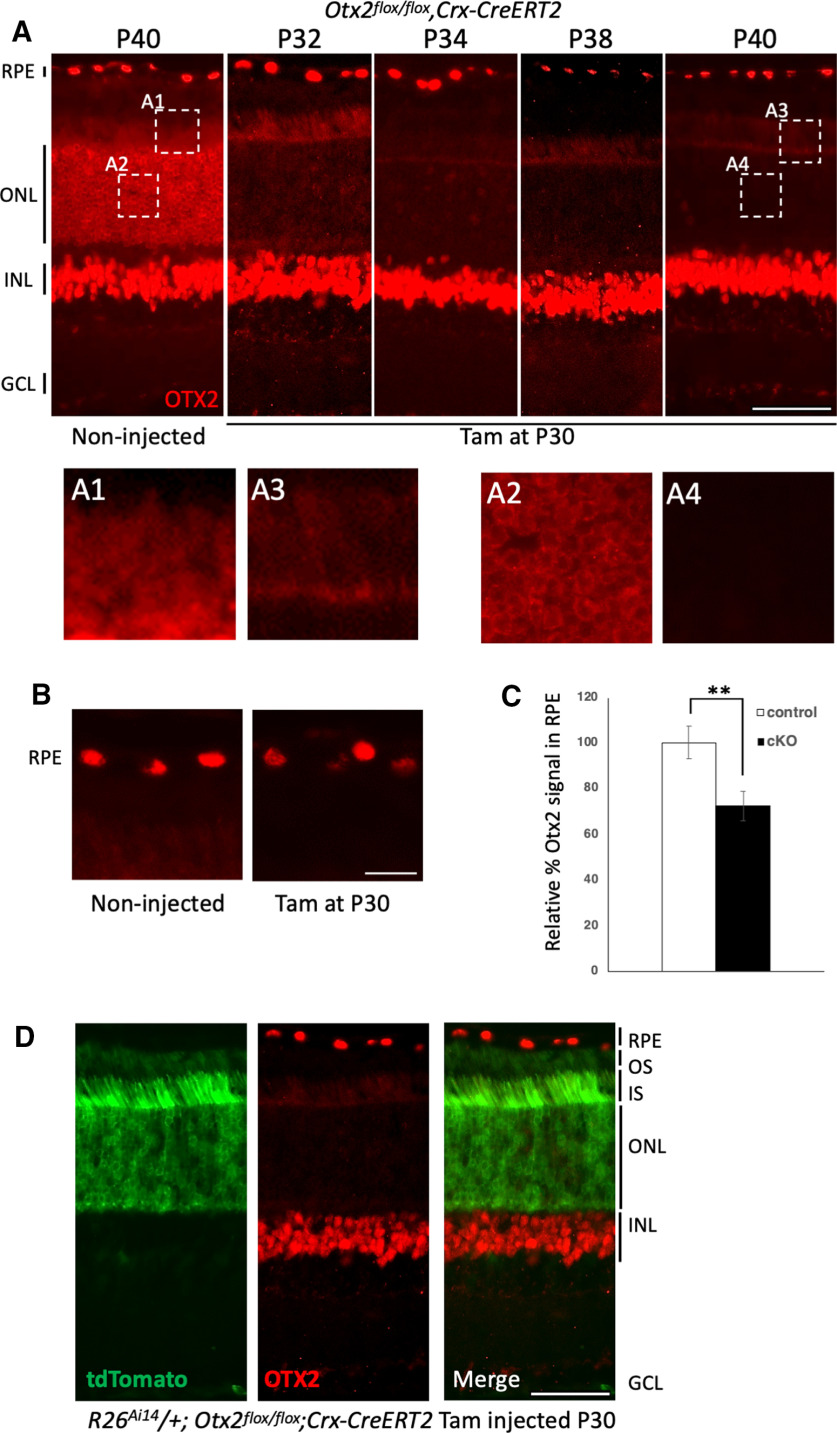
Otx2 specific ablation from PRs affects OTX2 protein level in the RPE. ***A***, Immunodetection of OTX2 in P40 control and P32, P34, P38, and P40 *Crx-CreERT2;Otx2^flox/flox^* cKO mice, tamoxifen-injected at P30. OTX2 is expressed in RPE, PRs (ONL), and BCs (INL). ***A1–A4***, Magnification of boxed ***A1–A4*** areas in ***A***. ***B***, Comparison of OTX2 signal intensity in RPE of P40 control and *Crx-CreERT2;Otx2^flox/flox^* cKO mice, tamoxifen-injected at P30. ***C***, Quantification of OTX2 relative signal intensity in the RPE of indicated mice. ***D***, Immunodetection of OTX2 in P90 control and tamoxifen-injected at P30 *Crx-CreERT2;Otx2^flox/flox^* mice. ***E***, Immunodetection of tdTomato (green) and OTX2 (red) in the retina of *Crx-CreERT2;Otx2^flox/flox^*;*R26^Ai14^* mice 10 d after injection. ONL, outer nuclear layer; INL, inner nuclear layer; OS, outer segments; IS, inner segments. Scale bars: 50 μm (***A***, ***D***, ***E***) and 10 μm (***A1–A4***, ***B***).

To achieve conditional ablation of *Otx2* in adult PRs, we crossed the *Crx-CreERT2* transgenic line (Muranishi et al., 2011) with *Otx2^flox/flox^* mice (Fossat et al., 2006), in which Cre-mediated recombination eliminates the second OTX2 coding exon, resulting in the production of a non-functional OTX2 N-terminal peptide that is not detected by OTX2 antibodies. Tamoxifen was administered at P30, and retinas were analyzed 2, 4, 8, and 10 d later (Fig. 1*A*). At P40, while fully maintained in BCs in the inner nuclear layer (INL), OTX2 protein could no longer be detected in nuclei of the outer nuclear layer of cKO mice (Fig. 1*A4*), showing efficient PR-specific *Otx2* ablation in the adult mouse retina. However, we noticed some OTX2 staining in the inner and outer segments of PRs in cKO mice (Fig. 1*A3*). Concomitantly, OTX2 showed weaker intensity in RPE of cKO mice than in RPE of control mice (Fig. 1*B*), suggesting a potential signal inhibiting OTX2 expression in the RPE or that the OTX2 protein found in inner and outer segments originated from the RPE. Quantification of the OTX2 signal in the RPE relative to the one in BCs (Fig. 1*C*) confirmed that OTX2 was decreased by 27% in the RPE of PR-specific *Otx2 c*KO compared with controls, supporting a putative movement of OTX2 protein from the RPE to PR segments. This decrease in OTX2 in the RPE appeared to be stable over several weeks after cKO induction (Fig. 1*D*).

Alternatively, the decrease of OTX2 in the RPE could be because of an ectopic expression of the *Crx*-*CreERT2* transgene. While no CRX expression in the mouse RPE has been described, it has been reported in bovine RPE (Esumi et al., 2009). We therefore explored the possibility of *Crx*-*CreERT2* activity in the RPE using the Cre-reporter *Gt(ROSA)26Sor^tm14(CAG-tdTomato)Hze^/J* (*R26^Ai14^*) line, which expresses the bright fluorescent tdTomato protein on Cre-mediated recombination (Madisen et al., 2010). *R26^Ai14/+^;Otx2^flox/flox^;CrxCreERT2* mice were tamoxifen injected at P30 and their retina were examined 10 d later (Fig. 1*D*). No tdTomato was observed outside PR cytoplasm and inner segments, ruling out the unwanted action of the *Crx-CreERT2* transgene in RPE cells. This lended weight to the hypothesis that PR-specific *Otx2* ablation depleted OTX2 protein from the RPE, thus reducing the amount of OTX2 remaining in RPE cells. We thus investigated the possibility of a transfer of OTX2 from RPE cells to PRs.

**Figure 2.**
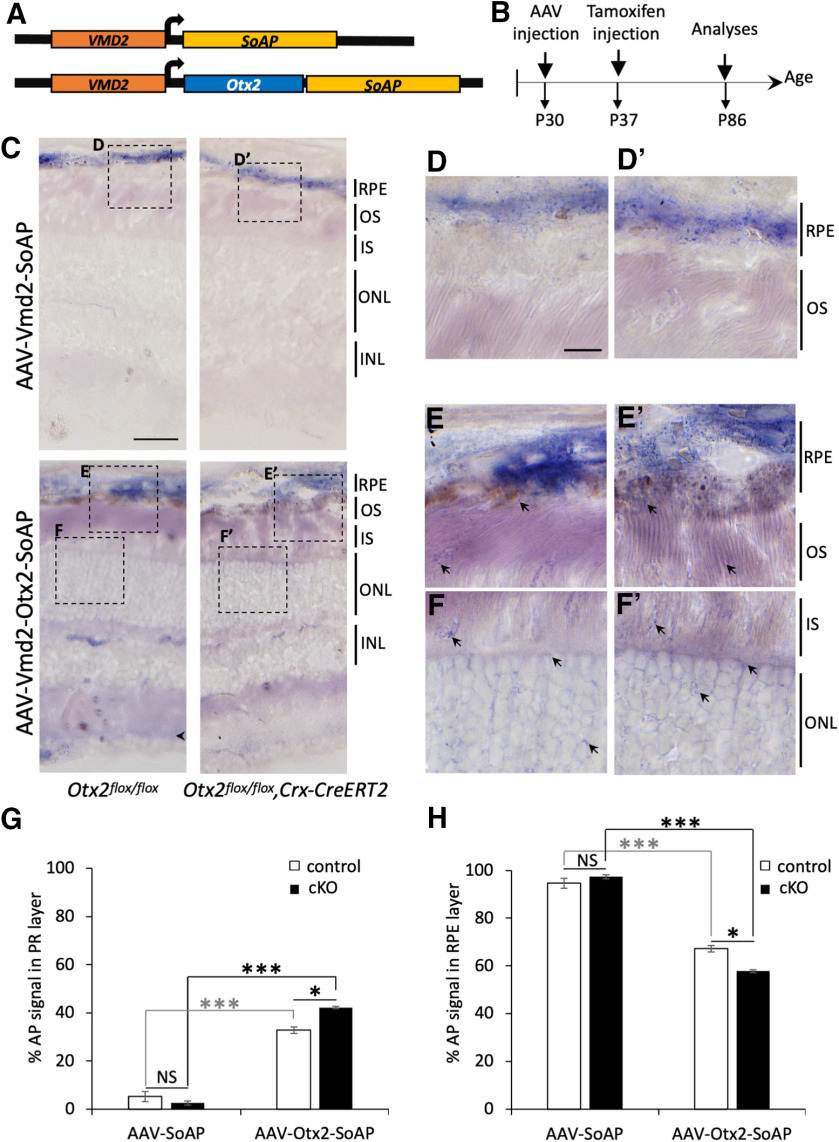
*In vivo* detection of OTX2 transfer from RPE to PRs. ***A***, Schematic structure of the constructs used for the production of AAV derived particles expressing SoAP-OTX2 or SoAP. ***B***, Schematics of the time course for AAV analysis. ***C***, AP staining of retinal sections from *Otx2^flox/flox^* and *Crx-CreERT2;Otx2^flox/flox^* eyes injected with the indicated vectors. ***D***, ***D’***, ***E***, ***E’***, ***F***, ***F’***, Magnification of boxed ***D***, ***D’***, ***E***, ***E’***, ***F***, ***F’*** areas in ***C***. Arrows highlight AP staining in internal regions of retina. ***G***, ***H***, Graph showing the relative signal intensities of AP in the PR layer (***G***) and RPE layer (***H***) of mice injected with indicated vectors (AAV-SoAP: *n* = 2; AAV-Otx2-SoAP: *n* = 3) and cKO (AAV-SoAP: *n* = 3; AAV-Otx2-SoAP: *n* = 2), expressed as a percentage of the total signal. For each mouse a minimum of three normalized fields of the region of interest per section and three different sections were used. In each normalized field, AP signal was determined using ImageJ by subtracting background signal from non-injected regions of the same eye. Error bars indicate SD *p <* *0.05, **p <* *0.01, ***p <* *0.005, statistically significant change (Student’s *t* test). OS, outer segments; IS, inner segments; ONL, outer nuclear layer; INL, inner nuclear layer. Scale bars: 50 μm (***C***) and 20 μm (***D***, ***D’***, ***E***, ***E’***, ***F***, ***F’***).

To distinguish OTX2 protein of RPE origin from other sources, we tagged OTX2 with SoAP, a modified version of the thermostable secreted placental alkaline phosphatase SEAP (Berger et al., 1988). As the presence of a secretion signal at the N-terminal end of a SEAP-OTX2 fusion protein could have influenced its location, we created SoAP a non-secreted version of SEAP, by deleting the seven-amino acid N-terminal signal peptide of SEAP. In addition, to limit interference with OTX2 addressing signals, the SoAP sequence was fused to the C terminus of OTX2 (Fig. 2*A*). To achieve RPE-specific expression at a physiological level, we made AAV vectors expressing SoAP or OTX2-SoAP fusion protein under control of the *Vmd2* promoter, which allows moderate, and very specific expression in the RPE (Guziewicz et al., 2013). AAV-SoAP and AAV-OTX2-SoAP were used for sub-retinal injection in P30 *Otx2^flox/flox^* control mice or *Otx2^flox/flox^; CrxCreERT2* mice. Tamoxifen was injected one week later, and retinas were collected six weeks later and processed for alkaline phosphatase (Fig. 2*B*). In AAV-SoAP-injected mice, SoAP was only found inside RPE cells in both control and cKO retinas (Fig. 2*C*). This indicated that the SoAP tag is neutral with respect to the localization of the proteins to which it is fused (Fig. 2*C*,*D*,*D’*). By contrast, in retinas injected with AAV-OTX2-SoAP, alkaline phosphatase staining could be detected in inner retinal regions encompassing the whole PR layer, with enhanced staining in retinas that had underwent *Otx2* ablation in their PR (Fig. 2*C*,*E*,*E’*,*F*,*F’*). Quantification of AP staining distribution between the PR (Fig. 2*G*) and RPE (Fig. 2*H*) compartments showed that while 95–100% of the AP signal was restricted to the RPE in AAV-SoAP injected control and cKO retinas, 32% of the AP signal was located in PRs in control retinas injected with AAV-OTX2-SoAP, and this proportion increased to 42% in cKO retinas.

Together, these results demonstrate a steady transfer of OTX2 protein of RPE origin to PR cells, the rate of which is increased after PR-specific ablation of *Otx2*.

**Figure 3.**
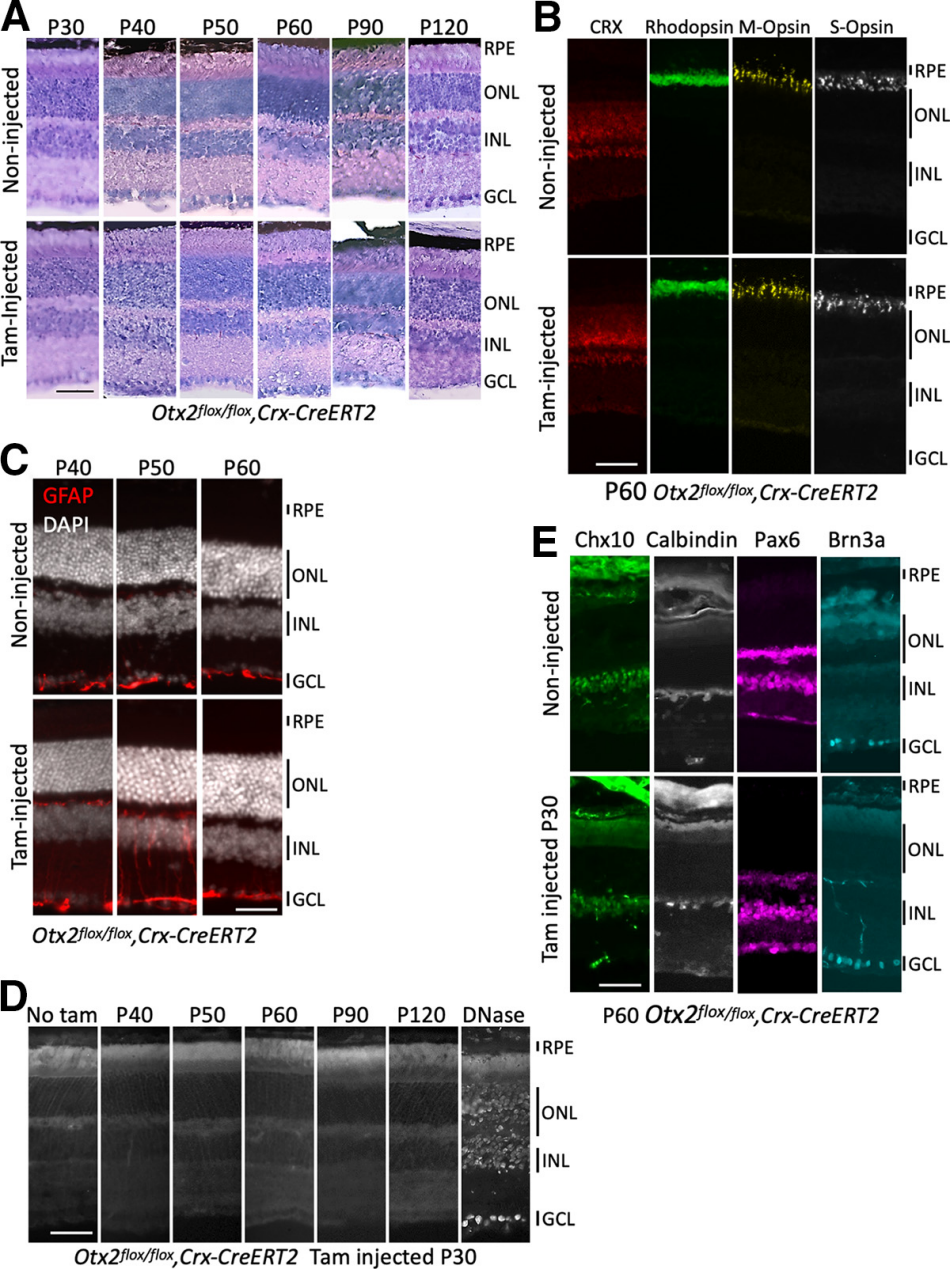
Otx2 expression in PRs is not required for cell viability or cell identity. ***A***, Hematoxylin/eosin staining of retinal sections of control *Crx-CreERT2;Otx2^flox/flox^* non-injected mice (upper panel) and cKO *Crx-CreERT2;Otx2^flox/flox^* mice, tamoxifen-injected at P30 (lower panel), at the indicated stages. ***B***, Expression of CRX, rhodopsin and M-opsin and S-opsin in P60 control (upper panel) and tam-injected at P30 (lower panel) *Crx-CreERT2***;***Otx2^flox/flox^* mice. ***C***, Expression of GFAP in control (upper panel) and tam-injected at P30 (lower panel) *Crx-CreERT2;Otx2^flox/flox^* mice at the indicated stages. ***D***, Detection of apoptotic cells by TUNEL staining of retinal sections of cKO mice 10 (P40), 20 (P50), 30 (P60), 60 (P90), and 90 (P120) days after tamoxifen injection (P30). Sections of mature retina (P32) were used as negative (left) and positive control (right, DNase treated). ***E***, Expression of cell-specific markers Chx10 (BCs), calbindin (horizontal cells), Pax6 (amacrine cells and RGCs), Brn3a (RGCs) in control upper panels and PR-specific Otx2 KO retinas (lower panels). ONL, outer nuclear layer; INL, inner nuclear layer; GCL, ganglion cell layer. Scale bars: 50 μm.

We then analyzed the long-term effect of *Otx2* ablation on retinal histology at P40, P50, P60, P90, and P120 (Fig. 3*A*). Contrary to what was shown in full *Otx2* KO or RPE-specific *Otx2* KO, which led to near-complete PR degeneration at P120 (Béby et al., 2010), retinal histology was preserved in PR-specific cKO mice. At P60, the general PR marker CRX and rod-specific and cone-specific opsins (Fig. 3*B*) showed no difference between control and cKO retinas, indicating normal PR maintenance and integrity. We next examined GFAP expression, a classical marker of retinal damage (Guerin et al., 1990). While in control mice, at P40, P50, and P60, GFAP only labeled astrocytes located in the nerve fiber layer of the retina, GFAP was upregulated in Müller cells in P40 and P50 cKO mice, indicating that in the absence of changes in retinal structure, PR-specific *Otx2* ablation induced transient and moderate retinal stress (Fig. 3*C*). However, no increased cell death was found (Fig. 3*D*) and specific markers for other neuronal cell types appeared normal (Fig. 3*E*). This confirms that *Otx2* expression in adult PRs is dispensable for the maintenance of retinal histology and integrity.

### PR-specific *Otx2* cKO causes light adaptation defects

While OTX2 does not appear to play a role in PR maintenance, its sustained expression throughout life in these cells suggests it might still be important for PR physiology and activity. During routine handling, we noticed that cKO mice tended to avoid bright light. To understand the origin of this behavior, we examined arrestin-1 movement, a protective mechanism that provides simultaneous information on PR response and on light adaptation (Gurevich et al., 2011). In the dark, arrestin-1 localizes in PR soma and inner segments. Bright illumination induces massive translocation of arrestin-1 to the outer segment, where it binds and inactivates rhodopsin, preventing an over-stimulation of the phototransduction cascade. When light is off, arrestin-1 moves back to other compartments of the cell, while transducin, the principal effector of phototransduction, moves in the opposite direction, leading to increased sensitivity of the PRs.

**Figure 4.**
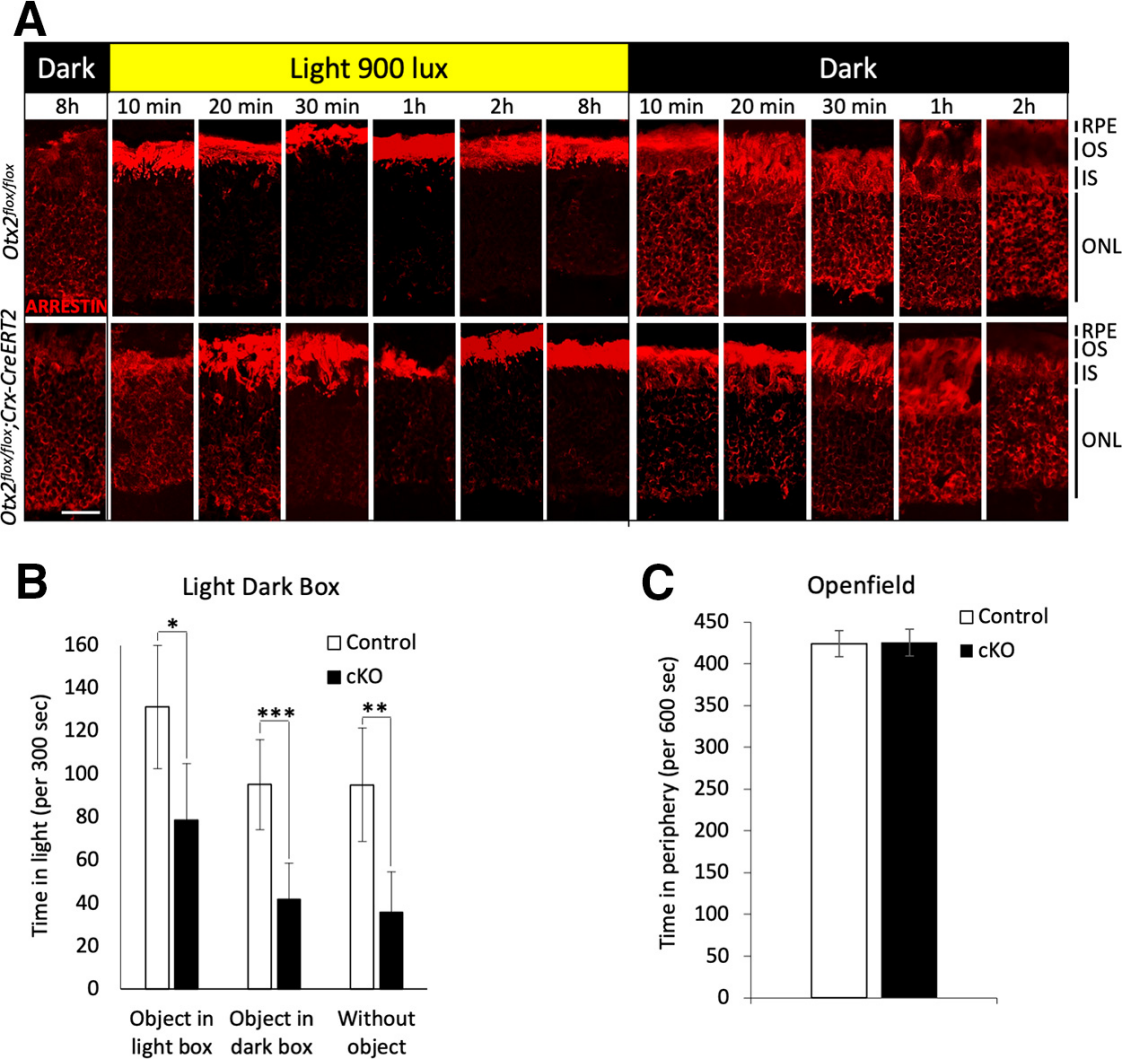
Arrestin-1 bidirectional translocation defect and light avoidance in cKO mice. ***A***, Kinetics of arrestin-1 translocation during light and dark adaptation: immunostaining of retinal sections of P50 mice showing arrestin-1 distribution 0, 10, 20, 30, 60, and 120 min after light onset (light 900 lux) and 0, 10, 20, 30, 60, and 120 min after light offset in control *Otx2^flox/flox^* (upper panel) and cKO *Crx-CreERT2;Otx2^flox/flox^* (lower panel) mice injected with tamoxifen at P30. ***B***, Light dark box test. Histograms represent the time spent (in seconds) in the light compartment in three conditions: with an attractive object in the light box (object in light box), with an attractive object in the dark box (object in dark box), or without any motivation (without object) in control (*n* = 10) and cKO (*n* = 13) mice (*p < 0.05, **p < 0.01, ***p < 0.005, *t* test). ***C***, Openfield test. Histograms represent the total time spent (in seconds) in the periphery of the openfield (*n* = 13; *p < 0.05, **p < 0.01, ***p < 0.005, *t* test). OS, outer segments; IS, inner segment; ONL, outer nuclear layer. Scale bar: 25 μm.

Dark-adapted mice were exposed to bright light and their retinas were collected after 10, 20, 30, 60, and 120 min of exposure. While in control mice, the vast majority of the arrestin-1 translocated to PR outer segments within 10 min, it took at least 30 min in cKO mice, and even after 120 min, some arrestin-1 was still visible in the outer nuclear layer (Fig. 4*A*). When light-adapted mice were put in the dark, arrestin-1 relocation began within 10 min in control mice, whereas it was again delayed until 60 min in cKO mice (Fig. 4*A*). Thus, in the absence of OTX2, arrestin-1 shuttling is slower in cKO mice, revealing a defect of adaptation to both light and dark in these animals.

To confirm that arrestin-1 translocation defect in cKO mice correlates with their light avoidance behavior, we performed preference tests using the light-dark box. As shown in Figure 4*B*, cKO mice spent significantly less time in the light compartment than control mice, even in the presence of a motivating factor such as the presence of an object. As this behavior could also be because of anxiety, we performed the open field test, which is sensitive to anxiety. In this test, cKO mice did not behave differently from control mice (Fig. 4*C*). We conclude that *Otx2* cKO mice are not more anxious than control mice but have an aversion to bright light.

### PR-specific *Otx2* ablation impacts ECM, cytoskeleton, signaling, and RPE-specific genes

**Figure 5.**
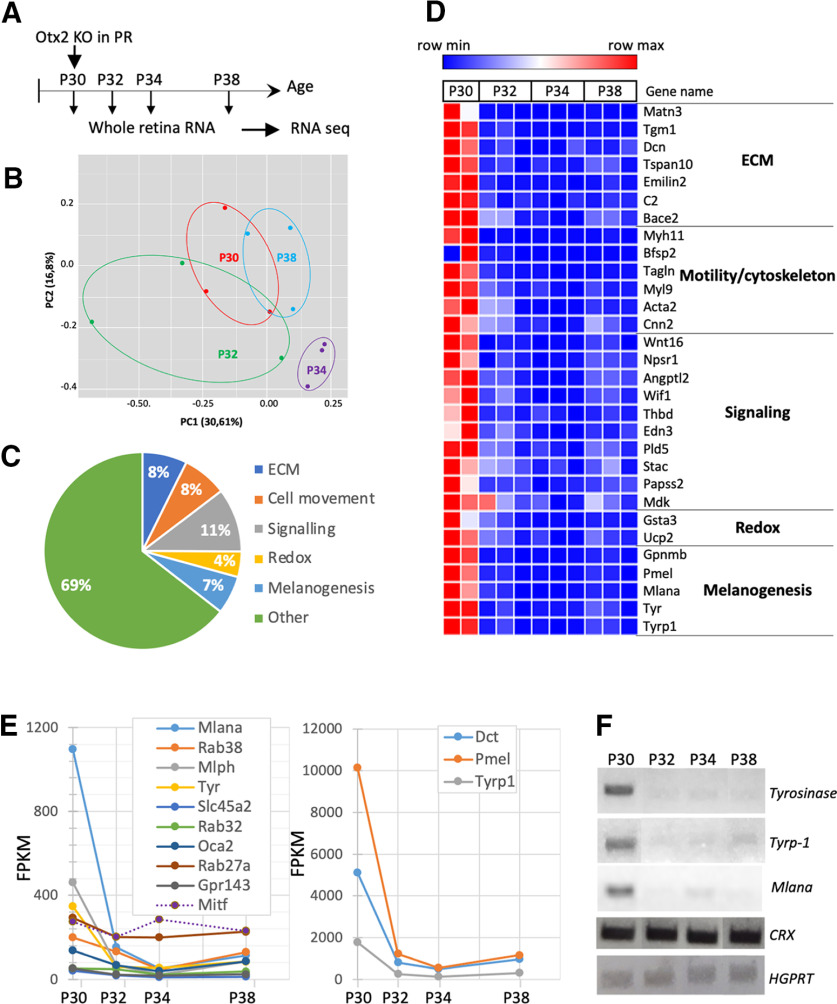
Time series transcriptomic analysis of PR-specific *Otx2* cKO retina. ***A***, Schematics of the protocol for time-series transcriptome analysis. ***B***, Principal component analysis. PCi axis represents the principal component i and the number into brackets indicates the percentage of explained variance associated with this axis. ***C***, Representative distribution of the functional groups of genes downregulated in the retina of PR-specific *Otx2* cKO mice 4 d after tamoxifen injection. ***D***, Heat-map representation of the variation of expression of assigned functional groups of downregulated genes, following *Otx2* cKO in PR. Gene expression (RPKM) was measured at the time of injection (P30) and 2, 4, and 8 d after KO (P32, P34, and P38). Color bar indicates the level of expression. ***E***, Temporal expression profile of selected genes. *y*-axis values represent an average of the replicates for each stage. Analyses were done in duplicate for P30 control mice (*n* = 2) and in triplicate for KO mice (*n* = 3) at the different stages. ***F***, Validation of RNA-seq data. Gene expression of three OTX2-target genes involved in RPE melanogenesis, *Tyrosinase*, *Tyrp-1*, and *Mlana* was evaluated by semi-quantitative RT-PCR. *CRX* and *HGPRT* were used as normalizers.

To identify short-term molecular changes induced by the conditional KO that might underlie the above phenotypes, we analyzed gene expression following PR-specific *Otx2* ablation. Since our previous full KO studies have shown that 2/3 of OTX2 target genes are downregulated 48 h after tamoxifen injection and all of them after 96 h (Housset et al., 2013), whole retina RNA, which includes RNA from RPE, was extracted at 0, 2, 4, and 8 d after tamoxifen injection at P30 and subjected to RNA-seq analysis (Fig. 5*A*,*B*). In the *Otx2^flox/flox^* mouse model, Cre-mediated recombination of the *Otx2* floxed allele removes the second OTX2 coding exon which encodes most of the homeodomain, but has no incidence on the level of *Otx2* transcript (Housset et al., 2013). To estimate deletion efficiency, we compared the number of reads of the floxed second coding exon (exon E5) relative to neighboring *Otx2* exon (E4) in control and cKO RNA samples (Table 1). A 28–32% relative reduction of reads matching the second coding exon was observed in cKO samples at P32, P34, and P38, consistent with full ablation in PRs and maintenance in RPE and BCs. Statistically significant deregulated genes (*p* < 0.05) were sorted according to their log2 fold change, for each time point (Table 2). Most were downregulated genes, with early and constant downregulation. Gene Ontology, ImPALA (impala.molgen.mpg.de), and GSEA (https://www.gsea-msigdb.org/gsea/index.jsp) software analyses of the genes downregulated at P32, P34, and P38 identified five functional groups (Fig. 5*C*,*D*). One group corresponded to ECM and IPM genes. Four days after *Otx2* ablation in PRs, we observed >50 downregulated genes related to ECM, with genes involved in chondroitin sulfate metabolism and ECM organization (collagens, fibronectins, glycosaminoglycans, and peptidoglycans protein core genes). The presence of *versican* (*Vcan*), *decorin* (*Dcn*), and *biglycan* (*Bgn*) in the list of downregulated genes indicated that IPM was also affected. These data suggested a role for OTX2 in the control of the three-dimensional network of extracellular macromolecules that provide biochemical and structural support to PRs and surrounding cells.

**Table 1:** Decrease of OTX2 exon 5 reads upon PR-specific cKO

**Timepoint**	**P30**			**P32**			**P34**			**P38**		
**Sample**	**1**	**2**	**3**	**1**	**2**	**3**	**1**	**2**	**3**	**1**	**2**	**3**
Reads_exon4	872	975	765	255	648	873	802	919	885	1156	1034	858
Reads_exon5	350	333	214	53	177	199	213	247	172	228	329	154
E5/E4 ratio	0.40	0.34	0.28	0.21	0.27	0.23	0.27	0.27	0.19	0.20	0.32	0.18
Mean E5/E4 ratio		0.34			0.24			0.24			0.23	
SD E5/E4 ratio		0.06			0.03			0.04			0.08	
% reduction E5		0.00			30.68			28.74			32.05	

Shown are absolute RNA-seq read numbers for the closest OTX2 exon 4 and floxed exon 5 at each timepoint. E5/E4 read ratios. mean and SD for each triplicate and the percentage of exon 5 reads reduction after cKO.

**Table 2:** Differentially expressed genes following PR-specific Otx2 invalidation.

2 days after			4 days after cKO			8 days after cKO		
Gene symbol	Log2 FC	Adjusted p-value	Gene symbol	Log2 FC	Adjusted p-value	Gene symbol	Log2 FC	Adjusted p-value
Upregulated genes								
Vmn1r15	1.085	4.223E-12	A330074K22Rik	0.691	5.520E-05	A330074K22Rik	0.940	2.771E-08
Frat2	0.721	7.028E-05	Pcdh9	0.513	5.035E-04	Cyp2j9	0.978	2.905E-05
1700030C10Rik	0.565	3.480E-03	Pcdh11x	0.645	1.163E-03	5930430L01Rik	0.741	7.131E-05
Dusp1	0.787	6.451E-03	Cdh12	0.619	1.738E-03	Ctif	0.562	7.917E-05
Nr4a1	0.506	1.435E-02	L1cam	0.531	1.987E-03	Tln2	0.575	1.435E-04
			Map1a	0.590	2.335E-03	Cdkl5	0.497	7.743E-04
			Kndc1	0.589	2.335E-03	Adcy1	0.520	2.169E-03
			Tusc5	0.607	2.944E-03	Hydin	0.783	3.502E-03
			Cdk14	0.500	4.562E-03	Fggy	0.736	1.005E-02
			Grin2a	0.552	5.688E-03	Faah	0.580	1.274E-02
			Caln1	0.515	7.558E-03	Wsb2-ps	0.761	1.275E-02
			Kif5a	0.519	7.810E-03	Spta1	0.505	1.358E-02
			Mctp1	0.560	7.987E-03	Mettl4	0.537	2.185E-02
			Cdkl1	0.568	8.289E-03	Kif5a	0.514	2.424E-02
			Rbfox1	0.518	8.492E-03	Map1b	0.497	2.424E-02
			Sik1	0.590	9.211E-03	Map1a	0.529	2.696E-02
			Lancl3	0.679	1.040E-02	Odc1	0.627	2.747E-02
			Hydin	0.656	1.077E-02	Lancl3	0.679	2.895E-02
			Srgap1	0.567	1.214E-02	Pcdh11x	0.545	2.939E-02
			Tmem151b	0.544	1.214E-02	Gm47021	0.570	3.193E-02
			Gpr158	0.505	1.214E-02	Cdh12	0.522	3.693E-02
			Pgr	0.598	1.415E-02	Impg1	0.502	3.766E-02
			Hecw2	0.512	1.446E-02	Gm5898	0.529	4.073E-02
			Ar	0.625	1.560E-02	Gm21955	0.723	4.788E-02
			Stc1	0.527	1.652E-02	Slc9a7	0.615	4.897E-02
			Kcnq3	0.538	1.909E-02	Srgap1	0.545	4.993E-02
			Atp2b3	0.553	2.078E-02			
			Tfap2d	0.547	2.141E-02			
			Htr5a	0.497	2.677E-02			
			Pcdha6	0.656	2.687E-02			
			Prlr	0.535	2.737E-02			
			D130079A08Rik	0.567	2.993E-02			
			Onecut3	0.541	3.183E-02			
			Ppp1r1c	0.590	3.270E-02			
			Gm8983	0.657	3.520E-02			
			Srl	0.540	3.528E-02			
			Pou4f1	0.501	3.590E-02			
			Ndst4	0.498	4.604E-02			
			Pcdha12	0.550	4.654E-02			
			Klf12	0.518	4.835E-02			
			Alk	0.502	4.851E-02			
Downregulated genes								
Myh11	-1.278	4.007E-11	Sod3	-1.744	5.984E-14	Myh11	-1.317	4.348E-10
Tagln	-1.151	2.092E-06	Vcan	-1.711	5.984E-14	Tgm1	-1.270	5.883E-08
Tgm1	-1.068	1.058E-05	Angptl2	-1.732	1.419E-13	Gsta3	-1.324	2.696E-07
Mlana	-0.980	1.372E-05	Penk	-1.675	6.155E-13	Tagln	-1.244	2.696E-07
Gpnmb	-1.020	1.588E-05	Cldn19	-1.640	1.474E-12	Myl9	-1.212	4.121E-06
Angptl2	-1.161	3.191E-05	Ltbp1	-1.507	1.474E-12	Rps3a1	-0.857	8.459E-06
Myl9	-1.157	3.191E-05	Myh11	-1.366	4.976E-12	Mlana	-1.028	1.104E-05
Wnt16	-0.902	3.191E-05	Dio3	-1.549	6.758E-11	Gpnmb	-1.035	2.905E-05
Pmel	-0.899	3.191E-05	Gsta3	-1.529	8.333E-11	Ubc	-0.886	2.905E-05
Matn3	-0.643	3.191E-05	Mrc2	-1.495	1.400E-10	Pmel	-0.930	4.049E-05
Emilin2	-1.056	5.366E-05	Col18a1	-1.429	1.400E-10	Matn3	-0.682	4.339E-05
4930435M08Rik	-0.730	5.366E-05	Tgm1	-1.375	1.661E-10	Emilin2	-1.079	6.306E-05
Gsta3	-1.118	6.722E-05	Serpine1	-1.452	1.398E-09	Wnt16	-0.881	2.147E-04
Rps3a1	-0.736	6.722E-05	Ism2	-1.423	1.948E-09	4930435M08Rik	-0.733	2.147E-04
Bfsp2	-0.513	1.099E-03	Efemp1	-0.840	1.948E-09	Bfsp2	-0.576	3.206E-04
C2	-0.994	1.115E-03	Bace2	-1.433	2.706E-09	Purg	-0.501	4.664E-04
Pld5	-0.948	1.405E-03	Tagln	-1.325	3.211E-09	Angptl2	-1.018	5.342E-04
Thbd	-0.979	1.908E-03	Matn2	-1.372	9.502E-09	Edn3	-0.965	5.886E-04
Npsr1	-0.974	1.935E-03	Myl9	-1.337	1.715E-08	Thbd	-1.002	6.007E-04
Tyrp1	-0.821	2.090E-03	Emp3	-1.369	1.769E-08	C2	-1.005	6.464E-04
Slc20a2	-0.569	2.809E-03	Edn3	-1.299	1.875E-08	Pld5	-0.919	7.127E-04
Tyr	-0.838	3.912E-03	Mlana	-1.162	2.164E-08	Acta2	-0.796	7.173E-04
Slc38a8	-0.921	4.018E-03	Atp1a2	-0.965	3.882E-08	Hpse	-0.888	1.152E-03
Bace2	-0.906	7.009E-03	Mgst1	-1.319	6.368E-08	Fam20a	-0.926	1.183E-03
Tspan10	-0.790	7.009E-03	Rarres2	-1.315	6.372E-08	Gm14268	-0.719	1.196E-03
Mlph	-0.753	8.764E-03	Slc38a8	-1.309	7.799E-08	Slc38a8	-0.962	1.365E-03
Ccdc3	-0.784	8.890E-03	Rhoj	-1.177	7.799E-08	Bace2	-0.949	2.010E-03
Dcn	-0.580	1.780E-02	Wnt16	-1.081	7.799E-08	Tyrp1	-0.838	2.109E-03
Lyz2	-0.809	1.841E-02	Gpnmb	-1.163	1.081E-07	4930458D05Rik	-0.928	2.169E-03
4932438H23Rik	-0.743	1.866E-02	Bgn	-1.248	1.155E-07	Shmt1	-0.867	2.169E-03
Acta2	-0.636	2.156E-02	Pmel	-1.054	1.384E-07	Slc43a1	-0.821	2.169E-03
Crybg1	-0.795	2.401E-02	Col9a2	-1.081	1.399E-07	Zfp566	-0.605	2.169E-03
Smtn	-0.800	2.734E-02	Fam129a	-1.292	1.437E-07	Smtn	-0.863	2.228E-03
Slc11a1	-0.811	3.130E-02	Crhbp	-1.289	1.474E-07	Adh1	-0.880	3.502E-03
Ucp2	-0.787	3.486E-02	Pld5	-1.180	1.894E-07	Ociad2	-0.762	3.928E-03
Papss2	-0.685	3.486E-02	Npsr1	-1.277	2.014E-07	AI464131	-0.722	3.970E-03
Palmd	-0.617	4.928E-02	Iqgap2	-1.155	2.153E-07	Npsr1	-0.878	6.593E-03
C130021I20Rik	-0.552	4.981E-02	Bmp2	-1.239	4.286E-07	Serpine1	-0.873	6.828E-03
			Wfdc1	-1.143	5.264E-07	Ctsk	-0.773	7.260E-03
			Emilin2	-1.190	5.997E-07	Papss2	-0.678	7.868E-03
			Col1a2	-1.179	6.514E-07	Gm44678	-0.857	8.088E-03
			Tmprss11e	-1.222	6.979E-07	Hbb-bs	-0.812	8.117E-03
			Rps3a1	-0.867	6.979E-07	Mlph	-0.754	8.948E-03
			Wif1	-1.064	7.023E-07	Gm38021	-0.855	8.998E-03
			Zic4	-1.206	9.790E-07	Tyr	-0.791	9.424E-03
			Acta2	-0.961	1.378E-06	Mmp14	-0.563	9.430E-03
			Zic1	-1.027	1.426E-06	Ucp2	-0.780	1.045E-02
			Mdk	-1.113	1.515E-06	Wdr95	-0.774	1.045E-02
			Notum	-1.189	1.515E-06	Gm26917	-0.636	1.045E-02
			Klhdc7a	-1.185	1.515E-06	Stac	-0.822	1.116E-02
			Dsg2	-1.172	1.999E-06	Cmtm3	-0.741	1.275E-02
			Gpx3	-1.148	2.441E-06	Wif1	-0.721	1.275E-02
			Matn3	-0.706	2.799E-06	Gxylt2	-0.637	1.275E-02
			Mecom	-1.159	2.908E-06	E230020A03Rik	-0.599	1.275E-02
			F5	-1.127	3.302E-06	Cnn1	-0.508	1.556E-02
			Clec18a	-1.030	3.797E-06	Dcn	-0.586	1.577E-02
			Tyrp1	-1.027	4.437E-06	Mdk	-0.749	1.623E-02
			Rab38	-1.134	6.774E-06	Dmgdh	-0.531	1.767E-02
			Defb9	-0.980	1.042E-05	Lrrc66	-0.791	1.808E-02
			Mlph	-0.986	1.224E-05	C130021I20Rik	-0.590	1.808E-02
			Thbs2	-1.115	1.283E-05	Gm28229	-0.583	1.872E-02
			Stac	-1.098	1.347E-05	Dpp4	-0.791	2.159E-02
			Ociad2	-0.938	1.347E-05	Slc24a5	-0.716	2.159E-02
			Ifi30	-0.886	1.368E-05	Gjb6	-0.609	2.250E-02
			Papss2	-0.874	1.374E-05	Palmd	-0.559	2.250E-02
			Gldc	-0.722	1.374E-05	Tspan10	-0.711	2.279E-02
			Idh2	-0.770	1.785E-05	Hbb-bt	-0.780	2.358E-02
			Lox	-1.083	2.474E-05	Pdgfc	-0.506	2.435E-02
			Cldn1	-1.055	2.533E-05	Nbl1	-0.540	2.465E-02
			Bfsp2	-0.596	3.016E-05	Nradd	-0.639	2.495E-02
			Ddr2	-1.029	3.228E-05	Gsx2	-0.643	2.747E-02
			Gfpt2	-1.012	3.228E-05	Uap1l1	-0.609	3.237E-02
			Smtn	-0.996	3.228E-05	Bgn	-0.721	3.273E-02
			C2	-1.076	3.230E-05	Eva1c	-0.738	3.356E-02
			Fbn1	-1.070	3.322E-05	Slc16a10	-0.589	3.356E-02
			Ucp2	-0.989	3.838E-05	Atp1a2	-0.538	3.397E-02
			Lyz2	-0.973	3.878E-05	Lox	-0.747	3.414E-02
			Thbd	-1.053	4.596E-05	Nkd2	-0.732	3.693E-02
			Ackr4	-1.054	4.779E-05	D130019J16Rik	-0.732	3.989E-02
			Cnn2	-1.046	4.779E-05	Selenop	-0.543	4.073E-02
			Fgfr1	-0.642	4.841E-05	Rny1	-0.556	4.975E-02
			Dse	-0.926	5.106E-05			
			D630039A03Rik	-1.053	5.266E-05			
			Nid2	-1.051	5.679E-05			
			Nkd2	-1.033	6.397E-05			
			Mrc1	-1.024	6.814E-05			
			C4b	-0.973	7.233E-05			
			Fbln7	-1.020	7.709E-05			
			Slc4a5	-0.722	7.939E-05			
			Pkdcc	-0.623	7.975E-05			
			Colec12	-1.025	7.996E-05			
			Aebp1	-0.978	8.145E-05			
			Tnfrsf21	-0.546	8.368E-05			
			Col9a1	-0.886	8.502E-05			
			Tyr	-0.955	8.525E-05			
			Zic2	-0.990	8.529E-05			
			Galnt12	-0.983	9.635E-05			
			Otx1	-0.967	1.012E-04			
			Sned1	-1.016	1.152E-04			
			Rapsn	-1.016	1.156E-04			
			Trip6	-0.676	1.282E-04			
			Mrgprf	-0.963	1.392E-04			
			Dio3os	-0.995	1.447E-04			
			Plin4	-0.980	1.541E-04			
			Igfbp4	-0.561	1.541E-04			
			Serping1	-0.968	1.576E-04			
			Gja1	-0.908	1.620E-04			
			Ces1d	-0.936	1.670E-04			
			Slc13a4	-0.929	1.715E-04			
			Dsg1a	-0.850	1.776E-04			
			BC067074	-0.992	1.884E-04			
			Fbln2	-0.985	1.913E-04			
			Crtap	-0.878	2.026E-04			
			Prelp	-0.604	2.104E-04			
			Tfeb	-0.581	2.148E-04			
			Cdk2	-0.819	2.214E-04			
			Suclg2	-0.933	2.246E-04			
			Col9a3	-0.779	2.465E-04			
			Ctsc	-0.612	2.465E-04			
			Adh1	-0.940	2.482E-04			
			Rcn3	-0.879	2.932E-04			
			Tspan10	-0.880	2.993E-04			
			Mdfic	-0.931	3.412E-04			
			Gm14268	-0.705	4.307E-04			
			H2-Aa	-0.947	4.931E-04			
			Ptgds	-0.932	5.035E-04			
			Bambi	-0.717	5.035E-04			
			Fam107a	-0.864	5.145E-04			
			Tst	-0.936	5.613E-04			
			Boc	-0.748	6.106E-04			
			4930458D05Rik	-0.921	6.644E-04			
			Slc26a4	-0.843	6.644E-04			
			Aldh1a3	-0.915	7.082E-04			
			2310034O05Rik	-0.891	7.269E-04			
			Timp3	-0.720	7.310E-04			
			Id3	-0.810	8.239E-04			
			Slc4a11	-0.912	8.411E-04			
			Perp	-0.913	8.428E-04			
			Ephx1	-0.845	8.534E-04			
			Fbn2	-0.899	1.040E-03			
			Zfp185	-0.855	1.065E-03			
			Slc24a5	-0.817	1.163E-03			
			Optc	-0.802	1.219E-03			
			F11r	-0.783	1.274E-03			
			Frrs1	-0.841	1.346E-03			
			Tgfbr3	-0.777	1.435E-03			
			Fbln1	-0.784	1.436E-03			
			Nbl1	-0.620	1.474E-03			
			Fkbp9	-0.766	1.621E-03			
			Veph1	-0.884	1.662E-03			
			Bmp4	-0.878	1.670E-03			
			1810022K09Rik	-0.502	1.670E-03			
			4930523C07Rik	-0.879	1.738E-03			
			Crocc2	-0.860	1.777E-03			
			Adamts9	-0.727	1.780E-03			
			Cald1	-0.549	1.798E-03			
			Selenop	-0.647	1.887E-03			
			Cd74	-0.850	1.906E-03			
			Olfml2a	-0.840	1.953E-03			
			Cyba	-0.715	1.953E-03			
			P3h1	-0.743	1.981E-03			
			Chmp4c	-0.869	2.023E-03			
			Clec14a	-0.848	2.158E-03			
			Tmem176a	-0.658	2.164E-03			
			P2rx6	-0.856	2.172E-03			
			Fmod	-0.805	2.172E-03			
			Slc16a12	-0.775	2.188E-03			
			Dcn	-0.622	2.188E-03			
			Piezo1	-0.834	2.313E-03			
			Gm7694	-0.684	2.313E-03			
			Tbx22	-0.762	2.335E-03			
			Gm44250	-0.857	2.391E-03			
			Flna	-0.647	2.391E-03			
			Gm26917	-0.644	2.567E-03			
			Ltbp2	-0.780	2.675E-03			
			Rapgef3	-0.627	2.798E-03			
			Nfatc4	-0.758	3.117E-03			
			Cmtm3	-0.753	3.245E-03			
			Arhgef5	-0.791	3.692E-03			
			Loxl3	-0.639	4.023E-03			
			Lamb2	-0.553	4.325E-03			
			Pon2	-0.635	4.345E-03			
			Rdm1	-0.646	4.497E-03			
			Cpz	-0.742	4.565E-03			
			Gypc	-0.818	4.607E-03			
			Nxn	-0.666	4.657E-03			
			Cyp4f15	-0.818	4.689E-03			
			H2-Eb1	-0.818	4.772E-03			
			Best2	-0.631	4.996E-03			
			Tlcd1	-0.581	5.262E-03			
			Blnk	-0.811	5.298E-03			
			Ccnd2	-0.793	5.298E-03			
			Ifitm2	-0.799	5.319E-03			
			Gas1	-0.508	5.477E-03			
			Eps8l2	-0.771	5.511E-03			
			Ctsh	-0.629	5.590E-03			
			Rhbdf1	-0.678	5.612E-03			
			Renbp	-0.583	5.768E-03			
			Eya1	-0.699	5.780E-03			
			Pon3	-0.803	6.065E-03			
			Ces5a	-0.682	6.065E-03			
			Ecm1	-0.651	6.164E-03			
			Pdgfrl	-0.736	6.211E-03			
			Efemp2	-0.526	6.239E-03			
			Ackr3	-0.762	6.363E-03			
			Acads	-0.560	6.473E-03			
			Cd82	-0.646	6.646E-03			
			Ankrd44	-0.601	6.646E-03			
			Tcim	-0.662	6.706E-03			
			Gpc4	-0.707	6.752E-03			
			Mfap4	-0.649	6.782E-03			
			C1s1	-0.787	7.613E-03			
			Lrig3	-0.611	7.810E-03			
			Agpat2	-0.755	7.846E-03			
			Tmem176b	-0.598	8.193E-03			
			Slc6a13	-0.728	8.241E-03			
			Fbln5	-0.781	8.253E-03			
			Apoe	-0.518	8.275E-03			
			Bdh2	-0.711	8.317E-03			
			AI464131	-0.622	8.474E-03			
			Cgnl1	-0.614	8.474E-03			
			Gm45407	-0.775	8.880E-03			
			Nectin3	-0.561	8.883E-03			
			Ehd2	-0.608	8.986E-03			
			Slc25a34	-0.745	9.339E-03			
			Dpp4	-0.771	9.344E-03			
			Ggt5	-0.769	9.344E-03			
			Pcolce	-0.756	9.344E-03			
			Gprc5c	-0.747	9.344E-03			
			Osgin1	-0.745	9.344E-03			
			Atp10d	-0.672	9.344E-03			
			Cobll1	-0.668	9.344E-03			
			Thap6	-0.575	9.826E-03			
			Gm38021	-0.767	9.845E-03			
			Slc45a2	-0.751	1.026E-02			
			1700055D18Rik	-0.708	1.065E-02			
			Slc11a1	-0.758	1.068E-02			
			Pear1	-0.736	1.073E-02			
			Htra1	-0.616	1.080E-02			
			Josd2	-0.534	1.083E-02			
			Shmt1	-0.697	1.098E-02			
			4930594M22Rik	-0.758	1.100E-02			
			Ahnak	-0.721	1.105E-02			
			Plekhg3	-0.516	1.105E-02			
			Slc22a8	-0.734	1.122E-02			
			Gm43637	-0.723	1.122E-02			
			Fcgrt	-0.714	1.122E-02			
			Dct	-0.633	1.214E-02			
			Nedd9	-0.544	1.214E-02			
			Stx11	-0.644	1.219E-02			
			Ctsk	-0.663	1.238E-02			
			Abcc6	-0.605	1.255E-02			
			Pdlim4	-0.673	1.273E-02			
			Fzd8	-0.545	1.342E-02			
			Tmem132b	-0.511	1.342E-02			
			Tmc6	-0.734	1.352E-02			
			Ptgr1	-0.718	1.363E-02			
			Cavin2	-0.733	1.372E-02			
			Syngr2	-0.667	1.372E-02			
			Gli2	-0.685	1.374E-02			
			Ager	-0.709	1.399E-02			
			Dok1	-0.736	1.419E-02			
			Pqlc3	-0.735	1.419E-02			
			Stab1	-0.708	1.419E-02			
			Plekhg2	-0.599	1.419E-02			
			AC152827.1	-0.640	1.447E-02			
			Echdc2	-0.574	1.460E-02			
			Tmem63a	-0.627	1.535E-02			
			Cd63	-0.550	1.619E-02			
			Ppfibp2	-0.683	1.626E-02			
			Vangl1	-0.603	1.651E-02			
			Slc2a1	-0.504	1.651E-02			
			Pgf	-0.724	1.652E-02			
			Kdelr3	-0.701	1.704E-02			
			Vstm4	-0.541	1.740E-02			
			A730049H05Rik	-0.720	1.751E-02			
			Hpse	-0.652	1.778E-02			
			Zic5	-0.698	1.785E-02			
			Wdr86	-0.681	1.785E-02			
			Sema3b	-0.611	1.785E-02			
			Pde3b	-0.516	1.785E-02			
			Sult1a1	-0.714	1.796E-02			
			Slc6a12	-0.626	1.892E-02			
			Itpripl2	-0.712	1.919E-02			
			Cldn2	-0.580	1.929E-02			
			Sqor	-0.709	1.957E-02			
			Iyd	-0.697	1.991E-02			
			Uap1l1	-0.570	2.070E-02			
			Mfrp	-0.669	2.118E-02			
			Abcc4	-0.655	2.118E-02			
			Gm42716	-0.680	2.132E-02			
			Nek8	-0.543	2.132E-02			
			Crybg1	-0.637	2.169E-02			
			C1qtnf6	-0.703	2.172E-02			
			Il17rc	-0.667	2.190E-02			
			Nradd	-0.577	2.303E-02			
			Mxra8	-0.682	2.333E-02			
			Tmem140	-0.697	2.336E-02			
			Stk26	-0.678	2.336E-02			
			1500015O10Rik	-0.593	2.336E-02			
			Aldh1a7	-0.684	2.360E-02			
			Antxr1	-0.611	2.368E-02			
			Vill	-0.694	2.394E-02			
			Megf6	-0.693	2.394E-02			
			Tspo	-0.654	2.471E-02			
			Slc35f3	-0.689	2.475E-02			
			Scube1	-0.675	2.481E-02			
			1600023N17Rik	-0.642	2.481E-02			
			Abi3bp	-0.684	2.495E-02			
			Ttc16	-0.667	2.519E-02			
			Fmo1	-0.640	2.519E-02			
			Gpr137b	-0.525	2.519E-02			
			Ano1	-0.606	2.708E-02			
			D630024D03Rik	-0.658	2.737E-02			
			Igfbp5	-0.563	2.741E-02			
			Gm42555	-0.683	2.747E-02			
			Trim63	-0.669	2.749E-02			
			Wtip	-0.537	2.848E-02			
			Fam20a	-0.641	2.956E-02			
			Ptpn14	-0.604	2.976E-02			
			Gli3	-0.537	2.976E-02			
			Stard8	-0.570	3.068E-02			
			Hes1	-0.504	3.086E-02			
			Folr1	-0.634	3.092E-02			
			Itpr3	-0.652	3.139E-02			
			Dll1	-0.526	3.183E-02			
			Tns1	-0.544	3.250E-02			
			Tnfrsf19	-0.586	3.266E-02			
			Gm30698	-0.646	3.269E-02			
			Mfap2	-0.629	3.269E-02			
			Hhip	-0.663	3.270E-02			
			Smco4	-0.655	3.270E-02			
			Fkbp10	-0.619	3.278E-02			
			Gm44037	-0.617	3.350E-02			
			1700124L16Rik	-0.576	3.404E-02			
			P4ha2	-0.560	3.410E-02			
			Rab32	-0.654	3.447E-02			
			Cavin1	-0.642	3.447E-02			
			Lrrk1	-0.657	3.479E-02			
			2610035F20Rik	-0.655	3.543E-02			
			Lama5	-0.611	3.543E-02			
			Pla2g4a	-0.654	3.590E-02			
			Gm25835	-0.651	3.590E-02			
			Aqp5	-0.655	3.618E-02			
			Ephb4	-0.614	3.625E-02			
			Shc1	-0.507	3.671E-02			
			Gsn	-0.606	3.685E-02			
			Timp1	-0.650	3.735E-02			
			Clec3b	-0.643	3.842E-02			
			Cldn7	-0.630	3.927E-02			
			2810030D12Rik	-0.508	3.927E-02			
			Lgals1	-0.597	3.940E-02			
			Igf2bp1	-0.561	4.000E-02			
			Ntf3	-0.594	4.039E-02			
			Ogn	-0.497	4.044E-02			
			Gm44678	-0.640	4.062E-02			
			Slc35g1	-0.608	4.064E-02			
			Ahcy	-0.634	4.097E-02			
			Slc39a12	-0.622	4.167E-02			
			Serpinh1	-0.642	4.169E-02			
			Rbm47	-0.636	4.205E-02			
			Maob	-0.582	4.210E-02			
			Tead2	-0.561	4.272E-02			
			Ptpn15	-0.610	4.275E-02			
			Slc4a4	-0.528	4.503E-02			
			Gje1	-0.510	4.511E-02			
			Rassf9	-0.633	4.516E-02			
			Six5	-0.579	4.559E-02			
			Gm20383	-0.606	4.573E-02			
			Plekhf1	-0.621	4.617E-02			
			Mboat1	-0.603	4.637E-02			
			Ch25h	-0.538	4.744E-02			
			Tmem150a	-0.514	4.753E-02			
			Snora73b	-0.628	4.835E-02			
			Wls	-0.580	4.841E-02			
			Adamts2	-0.577	4.841E-02			
			Loxl1	-0.610	4.858E-02			
			Gm12689	-0.620	4.936E-02			

**Table 3.** Statistical table

**Data structure**	**Type of test**	**Power**
Figure 1*C*		
Relative % OTX2 signal in RPE	t-test (two-way ANOVA with repeated measures)	0.98
Normal distribution		
Figure 2*G*		
%AP signal in PR layer	t-test (two-way ANOVA with repeated measures)	
ct + AAV-SoAP vs cKO + AAV-SoAP		n.s
ct + AAV-Otx2-SoAP vs cKO + AAV-Otx2-SoAP		0.97
ct + AAV-SoAP vs ct + AAV-Otx2-SoAP		0.99
cKO + AAV-SoAP vs cKO + AAV-Otx2-SoAP		0.99
Figure 2*H*		
%AP signal in RPE layer	t-test (two-way ANOVA with repeated measures)	
ct + AAV-SoAP vs cKO + AAV-SoAP		n.s
ct + AAV-Otx2-SoAP vs cKO + AAV-Otx2-SoAP		0.97
ct + AAV-SoAP vs ct + AAV-Otx2-SoAP		0.99
cKO + AAV-SoAP vs cKO + AAV-Otx2-SoAP		0.99
Normal distribution		
Figure 4*B*		
Light-Dark Box test	t-test (two-way ANOVA with repeated measures)	
Object in light		0.99
Object in dark		0.99
Without object		0.99
Normal distribution		
Figure 4*C*		
Openfield test	t-test (two-way ANOVA with repeated measures)	n.s
Normal distribution		

Another group contained the genes coding for Myosin heavy chain 11 (*Myh11*), Beaded Filament Structural Protein 2 (*Bfsp2*), Transgelin (*Tagln*), Myosin light chain 9 (*Myl9*), Actin α 2 (*Acta2*) and Calponin 2 (*Cnn2*). This suggests a coordinated decrease of components of the actin cytoskeleton following *Otx2* cKO in PRs, which could explain the arrestin-1 translocation defect. The third assigned group of downregulated genes comprised genes involved in signaling such as *neuropeptide S receptor 1* (*Npsr1*), *angiopoietin-like 2* (*Angptl2*), *thrombomodulin* (*Thbd*), *phospholipase D5* (*Pld5*), and *3'-phosphoadenosine 5'-phosphosulfate synthase 2* (*Papss2*) and genes related to TGF-ß and Wnt signaling, such as *Wnt16* and *Wnt inhibitory factor 1* (*Wif1*). Among downregulated genes, especially at P34, were also genes with oxido-reductive functions. Some of these encode cytochrome subunits, with a mitochondrial localization, involved in the respiratory chain and playing a role in ATP production. Other downregulated oxidoreductases were the mitochondrial amidoxime reducing component 2 (*Mtarc2*) and the Fatty acyl-CoA reductase 2 (*Far2*). The fifth assigned group corresponded to genes involved in melanogenesis, including *Tyrosinase*, *Tyrp1*, *Slc45A2*, *PMEL* (*Silver*), and *Mlana* (Fig. 5*D*,*E*). This was unexpected because all these genes are expressed in the RPE, but not in PR cells where *Otx2* cKO had taken place. However, all of them were previously shown to be OTX2 direct targets in the RPE (Housset et al., 2013) and to have OTX2 ChIP-Seq peaks (Samuel et al., 2014). Their downregulation, confirmed by semi-quantitative RT-qPCR for *Tyr*, *Trp1*, and *Mlana* (Fig. 5*F*), nicely fits with the enhanced transfer of OTX2 toward PR segments and its parallel depletion of OTX2 from RPE on PR-specific *Otx2* cKO (Fig. 1*A*,*B*). This appears to be highly specific, as other RPE-specific genes like *Mitf*, also involved in melanogenesis but not under the control of Otx2, were not altered (Fig. 5*E*). The strong downregulation of these RPE-specific OTX2 target genes following PR-specific *Otx2* ablation confirmed that OTX2 activity was decreased in RPE cells.

## Discussion

Using cell-specific KO to study the function of OTX2 in adult PR cells, we revealed new functions of PR OTX2 as well as the existence of a transfer of OTX2 from RPE to PR cells, possibly associated to neuroprotective functions.

### *Otx2* cKO does not affect retina integrity

*Otx2* cKO restricted to PRs neither affects cell viability nor identity. Although a switch of cell identity is not frequent, this can occur on gain or loss of transcription factors. For instance, ectopic expression of *Pax4* can reprogram α or δ-pancreatic cells into β-cells (Collombat et al., 2009; Chung et al., 2010; Druelle et al., 2017). In the developing retina, *Otx2* KO respecifies PR precursors into amacrine precursors (Nishida et al., 2003; Yamamoto et al., 2020). In the adult retina, cell identity no longer requires OTX2, indicating that identity is robustly established. We did not find a role for OTX2 in major PR cell-specific functions; levels of opsins or of phototransduction proteins such as arrestin-1 were unchanged. This could be because of CRX expression in PR, which could compensate for the loss of OTX2. Both proteins recognize the same DNA motif (Chen et al., 1997; Chatelain et al., 2006), have overlapping genome occupancies (Samuel et al., 2014) and thus, could substitute for each other. A way to reveal OTX2 and CRX shared target genes in PRs would be to KO *Crx* simultaneously to *Otx2* in adult PR cells. Unfortunately, no conditional *floxed-Crx* allele is available. Somatic *Crx* ablation with CRISPR/Cas9 in future studies might help solve this issue.

### Arrestin-1 translocation defect and photophobia

*Otx2* KO in PRs causes an arrestin-1 translocation defect associated with photophobia. The defect concerns both directions of translocation. Arrestin-1 movement (Broekhuyse et al., 1985) is part of a process of protein translocation between PR compartments triggered by lighting changes (Strissel et al., 2006). It is thought to contribute to rod maintenance. Its main function would be to protect PRs against phototransduction signaling overload during the day and spare them for dusk (Gurevich et al., 2011).

Arrestin-1 forms oligomers (Kim et al., 2011). The translocation delay we observe here is similar to that seen in mice expressing oligomerization defective arrestin-1 mutants (Samaranayake et al., 2020). While *Otx2* cKO is unlikely to affect the formation of oligomers, as this process is autonomous (Kim et al., 2011), it might affect the energy supply needed to control oligomers movement (Satoh et al., 2010). Energy requirement for arrestin-1 translocation is debated (Mendez et al., 2003; Nair et al., 2005; Slepak and Hurley, 2008), but considering the cost of active transport, diffusion is believed to account for most of arrestin-1 movement (Gurevich et al., 2011). Nevertheless, the control of translocation by light needs a gating energy (Satoh et al., 2010). Since each PR consumes ∼10^8^ ATP.s^−1^ to support the Na^+^ influx through cGMP-gated channels, plus the ATP needed to control light-induced protein translocation (Okawa et al., 2008), any small variation in ATP production is likely to affect this process. OTX2, which stimulates mitochondrial energetics and ATP level in BCs (Kim et al., 2015), could influence the level of energy necessary to control arrestin translocation.

Arrestin-1 translocation is also affected by inhibitors of actin polymerization (Peterson et al., 2005; Reidel et al., 2008), indicating that this process requires functional microfilaments. A role of OTX2 in the control of cytoskeleton genes has long been proposed (Boncinelli and Morgan, 2001). The downregulation of myosin light and heavy chains, actin, calponin, and other partner genes that we observe on PR-specific *Otx2* cKO confirms that in adult PR, OTX2 coordinates the expression of major actors of the actin cytoskeleton. Together, defects in ATP production and microfilament in *Otx2* cKO retinas may contribute to the arrestin-1 translocation defect.

### PR-specific *Otx2* ablation causes downregulation of IPM and ECM genes

*Otx2* ablation in PRs affects the expression of IPM and ECM genes. The ECM is made of collagen, elastin, and carbohydrates assembled into a meshwork with structural and signaling roles (Kular et al., 2014; Alisafaei et al., 2021). The specialized IPM, which fills the space between PRs and RPE, is devoid of collagens and elastin (Ishikawa et al., 2015). Chondroitin sulfate proteoglycans (CSPGs) are abundant in both matrices, but versican, decorin, and biglycan are specific components of the IPM (Ishikawa et al., 2015). In the visual cortex, OTX2 binds to CSPGs of perineuronal nets (PNNs) surrounding parvalbumin cells (Beurdeley et al., 2012). CSPGs interact with signaling molecules and facilitate their interaction with target cells (Mizumoto et al., 2013). In parvalbumin cells, OTX2 binding to PNN promotes its internalization and subsequent maturation of the PNN (Beurdeley et al., 2012; Bernard and Prochiantz, 2016). The regulation of ECM and IPM genes by OTX2 in the mature retina observed here suggests that this could be a general function of OTX2 in neurons.

The ECM participates in tissue rigidity and signaling activities. This may explain our observation that *Otx2* mutant retinas tend to tear more easily than control retinas. The fact that more OTX2 secreted by the RPE is internalized by PRs after *Otx2* cKO may also reflect a modification of signaling activities. In the visual cortex, PNN disruption reduces OTX2 binding and internalization specificity (Bernard and Prochiantz, 2016). Similarly, downregulation of CSPGs following *Otx2* KO in PRs could facilitate the transfer of extracellular OTX2. The IPM could contain components secreted by PR cells under the control of OTX2, that limit OTX2 diffusion. Following *Otx2* KO in PR cells, their concentration would decrease, allowing facilitated diffusion of RPE-derived OTX2, and possibly of other factors. A thorough analysis of IPM composition in control and *Otx2* PR-specific KO retinas could reveal whether IPM contains such components and help identifying the signaling factors that PR and RPE cells exchange.

### OTX2 transfer from RPE to PR

The decrease of RPE OTX2 and the concomitant downregulation of RPE-specific target genes following PR-specific *Otx2* cKO led us to highlight a transfer of OTX2 protein from the RPE to PR cells that has not been described before.

What makes OTX2 basal transfer increase in PR cKO retina? We see two non-exclusive possibilities: either *Otx2* cKO PRs actively send a signal to the RPE, and/or the increased transfer is passive. The increase of GFAP seen in Müller cells following cKO could reflect and even contribute to an active signal. On the other hand, the existence of a basal transfer of OTX2 from the RPE to the PRs sets the stage for a passive effect: after *Otx2* cKO in PR cells, the decreased endogenous OTX2 concentration could simply create an imbalance. Together with the downregulation of IPM-specific proteins, which might facilitate diffusion of OTX2, this could increase the transfer rate and restore the equilibrium.

Since the serendipitous discovery of homeodomain internalization (Joliot et al., 1991), intercellular homeoprotein transfer has been found in several organisms and tissues (Di Nardo et al., 2020) and concerns many homeoproteins (Lee et al., 2019). Besides their cell-autonomous functions as transcription factors, homeoproteins, among which OTX2, PAX6, VAX1, and Engrailed can exert other functions outside of their producing cells, such as signaling, maturation and neuroprotection (for review, see Di Nardo et al., 2018). Engrailed was shown to contribute to RGC axon guidance by stimulating ATP synthesis (Stettler et al., 2012). A similar role was found for exogenous OTX2 in T2 OFF-BCs (Kim et al., 2015), further supported by a proteomic study showing OTX2 association with mitochondrial proteins involved in energy production (Fant et al., 2015).

What could be the role of transferred OTX2 in PR? Until recently, functional analysis remained difficult, because the motifs controlling secretion and internalization lie within the homeodomain, making cell-autonomous and non-cell-autonomous activities impossible to separate by genetic mutations (Di Nardo et al., 2018). However, recent studies using secreted single-chain OTX2-antibodies to trap extracellular OTX2 have made it possible to investigate non-cell-autonomous functions without interfering with cell-autonomous ones (Bernard et al., 2016). In the retina, this strategy demonstrated the importance of extracellular OTX2 for internal retinal activity and visual acuity (Torero Ibad et al., 2020). To probe the role of exogenous OTX2 of RPE origin, one possibility would be to conditionally express single-chain OTX2-antibodies in PRs with or without simultaneous deletion of the endogenous *Otx2* gene. OTX2 neuroprotective role could then be estimated by assessing PR fitness.

In conclusion, we have found a new level of inter-cellular interaction in the retina, that relies on OTX2 transfer from RPE to PR cells. There seems to be a directionality of the transfer: the proposed source of OTX2 found in RGC was PR or BC (Sugiyama et al., 2008). The source of OTX2 in T2-OFF BC is PR, not BC (Kim et al., 2015). Here, the source of OTX2 we find in PR outer segments and cytoplasm is RPE. This evokes a generalized inward transfer of OTX2, that might reflect a level of close proximity interactions essential for the physiology and long-term maintenance of the retina.

## Synthesis

Reviewing Editor: Susanne Wolf, Charite University Hospital Decisions are customarily a result of the Reviewing Editor and the peer reviewers coming together and discussing their recommendations until a consensus is reached. When revisions are invited, a fact-based synthesis statement explaining their decision and outlining what is needed to prepare a revision will be listed below. The following reviewer(s) agreed to reveal their identity: NONE.

Both reviewers found the paper interesting to the eNeuro community. Howerver, they raised major concerns especially about data presentation and method description. The specific comments and questions are listed below.

Reviewer 1

The authors investigated the cell-autonomous effects of a tamoxifen-induced photoreceptor conditional Otx2 knockout (cKO) in mature mouse retina. Immunohistochemical characterization of the retina indicated that the overall morphology and major cell types were normal, in contrast to the photoreceptor degeneration in a retinal pigmented epithelium (RPE)-specific Otx2 KO. Despite the loss of photoreceptor nuclear Otx2 in the photoreceptor cKO, Otx2 immunoreactivity in the outer nuclear layer (ONL), inner segment, outer segment was observed. Using an alkaline phosphatase (AP)-tagged Otx2 (AAV-Vmd2-Otx2-SoAP) expressed only in RPE, they showed that this unexpected Otx2 came from the RPE. As this is a key finding of this paper, more technical details are needed, e.g. what steps were done to ensure that the staining in the control and cKO developed equally. In general, the method used to quantify the staining intensities needs to be described more fully, e.g., how was the intensity of the NBT/BCIP reaction product determined in ImageJ, what was the region of interest (ROI) used, how was the normalization of the staining intensity measurements done, etc. Using behavioral assays, the PR Otx2 cKO mice demonstrated increased light avoidance over controls. The translocation of arrestin in these Otx2 cKO PRs showed a slower kinetics than did controls to both light and dark adaptation. However, the correlation (P.12) between the slowed arrestin translocation and light avoidance behavior is not a very direct one, especially as they show many classes of genes were down regulated, as identified by RNA-seq analysis performed on the tamoxifen-induced PR Otx2 cKO retinas at different post-injection time points. The reduction in genes involved in melanogenesis in RPE is consistent with the reduced Otx2 levels observed in their AAV-Vmd2-Otx2-SoAP assay.

Major

1. The drop in Otx2 staining in RPE with the presence of Otx2 in the photoreceptors in the cKO is cited as evidence for the transfer of Otx2 from RPE to the photoreceptors. The quantification appears to support this observation, but the unevenness of the AP-NBT/BCIP reaction product and the disrupted tissue in the images in Figure 2 E, E', F, F' prompt questions of how the quantification was done. What was taken for the regions of interest (ROI) for the various areas?

2. More details of how the images were taken are needed. For the confocal images, are they projections? In Figure 4, were all of the retina sections from the different time points processed for immunohistochemistry at the same time and imaged under equivalent settings? The ONL in the 30 min Dark panel for the cKO appears significantly less intensely stained for arrestin. The IS/OS/RPE layers in the 1 h Dark panels for control and cKO appear disrupted compared to the other sections. Are the non-GCL immunostaining for Brn3a and non-INL staining for Chx10 non-specific staining artifacts?

3. The AP-NBT/BCIP reaction product appears to be cytoplasmic in the ONL in Figure 2. Is there a molecular difference in the Otx2 found in the nucleus vs. that found in the cytoplasm?

4. The slowed kinetics of arrestin translocation from minutes to hours between photoreceptor compartments may underlie light adaptation defects. The sentence in the discussion (p.15, 2nd paragraph) however, regarding light adaptation taking seconds with the Calvert et al., 2006, citation (although the Gurevich et al., 2011, would appear to be the more accurate citation) undercuts this argument.

5. Do the control and cKO mice behave similarly in the Light-Dark Box or Open field tests after 2-8 h in light, or after 2 h in dark, when the arrestin distributions are similar? Presumably the mice start off light adapted, before the 30 min in the dark before testing. Is that so?

6. Are the genes that are down regulated following PR Otx2 cKO presumed to be primarily in photoreceptors and RPE? Can some of the gene expression changes occur in bipolar cells, some of which are thought to obtain Otx2 from photoreceptors?

Minor

1. The amount of tamoxifen injected for the behavioral experiments is double from that stated in the Animals section. Was there a reason for this?

2. Antibody information for calbindin, Pax6 and Brn3a antibodies is needed.

3. The graphs in Figure 2 in G and H are switched from what is in the text.

4. The figure legend of 4 describes quantification (E,F) that is not in the final figure, and are the scale bars for panel A? Scale bars are listed for C (a graph) and D (missing).

5. P.13, 2nd paragraph: TGF-β

6. The references are out of alphabetical order.

7. Journal name is missing in the Nishida et al. (2003) reference.

8. P.5, bottom of the page: Does “time spent ... all over the test” means the “for the whole duration of the test”?

Reviewer 2

In this manuscript entitled “Photoreceptor cKO of OTX2 enhances OTX2 intercellular transfer in the retina and causes photophobia", the authors studied the cell-autonomous function of the homeoprotein OTX2 in PRs by deleting specifically Otx2 in adult rod and cone PRs using Crx-CreERT2. Despite loss of OTX2 expression in the ONL after tamoxifen injection at P30, the protein was still found in PR segments suggesting a transfer of OTX2 from the RPE to PRs. They verified the ability of OTX2 to transfer from the RPE to the PRs using an OTX2-expressing AAV vector with a RPE-specific promoter. Lack of OTX2 in PR did not alter retinal structure but impaired the translocation of Arrestin-1 making mice photophobic. RNA-Seq analysis revealed down-regulation of set of genes involved in pathways related to cytoskeleton, extracellular matrix, signaling, and melanogenesis that might account for the observed phenotype and the enhanced transfer of OTX2.

This manuscript is well written and data are quit convincing however several issues / questions listed thereafter should be addressed before publication.

One of the key data of this manuscript is the remaining detection of OTX2 in the PR segments associated with a decrease of OTX2 expression in RPE despite the cKO of OTX2 specifically in PR. In the result part, they directly hypothesize that this result is due to OTX2 transfer from RPE. They should moderate/temper the hypothesis by also mentioning a potential signal inhibiting OTX2 expression in RPE (as written in the discussion).

They claim that CRX expression has not been reported in RPE. However, the IHC from the reporter line Ai14 with Crx-CreERT2 does not show a clear signal in the inner nuclear layer where CRX expression was reported (Glubrecht 2013; Yamamoto 2019; Nishida 2003) (A weak signal may be present). It is not clear in the manuscript why the authors decided to use an anti-TdTomato to visualize where the Crx-CreERT2 was expressed. On OCT sections, the fluorescence could be directly visualized? Can the authors definitively rule out the absence of recombination in cKO Otx2 Crx-Cre RPE by doing PCR on isolated RPE? They could use a set of primers able to detect the presence or not of the recombination for the second OTX2 coding exon.

Concerning the decrease of Otx2 expression in RPE following OTX2 deletion in PR, the authors performed a quantification based on fluorescence signal intensity. For better quantification, immunoblot analysis on isolated RPE should be performed. Finally, they checked OTX2 expression in PR and RPE at only a one-time point (10 days after tamoxifen injection). Is the decrease of OTX2 expression in RPE maintained during aging? Is the inhibition increased over time?

Concerning the phenotype of cKO OTX2 mice, the authors did not mention ERG recordings. Did they perform photopic and scotopic ERGs? Is the flicker ERG altered?

Did the authors check retinal integrity after P120? PR degeneration could be much slower than observed in RPE Otx2CKO animals.

At P60 GFAP signal seems to be decreased. Is it true or the selected picture showed in the manuscript is not representative? Did they quantify by qPCR GFAP activation? If it is less activated it could suggest a compensatory mechanism.

Concerning the data on protein trafficking, the authors should provide pictures of transducin mislocalization/relocalization kinetic to confirm Arrestin 1 data. As they mention in the introductory sentence, these two proteins have opposite movement during the dark-light transition.

The authors performed RNA-Seq analysis to identify short-term molecular changes induced by the conditional knockout that might underlie the observed photophobia. In that case, why did they kept the RPE for RNA-Seq analysis?

They estimated a 28-32% decrease of reads matching the second coding exon as early as 2 days post-injection. Did they estimate this change by counting directly reads without library size normalization? If it is the case, they can make read counting at exon-level and provide CPM values for this specific exon at each time point.

Even if deletion occurs rapidly, they need to provide an IHC with anti Otx2 antibody to visualize the kinetic of the protein loss in the ONL up to 8 days postinjection. Immunoblot analysis might also be sensitive enough. It is quite striking how fast the downregulation of the discussed genes occurs. Did the authors check that tamoxifen injection in cKO OTX2 without CRE has no impact on the expression of the discussed genes? Did they find downregulated genes after two days but unchanged between the time of injection and day 2? Could the authors provide a PCA of the different samples to visualize when the highest difference occurs during the kinetic? Along the same line, authors should also provide a Table with FC and p-value to have an idea about the amplitude of the changes and mention how many differentially expressed genes were found for each comparison.

The expression values showed in Fig 5D and E correspond to what? CPM? TPM? FPKM? Z-score? The author should make a heatmap using TPM to take into account gene length and depth of the sequencing for fig5D. Heatmap based on z-score could also be provided. Fig5E should also be done based on TPM expression, which is now commonly used for gene expression analysis.

Can the authors provide the list of the “few” upregulated genes? What are they? Do they belong to specific pathways?

Authors found RPE-expressed downregulated genes. Since they already published ChIP-Seq from cKO-OTX2 in RPE, it would be interesting to separate the downregulated genes with Otx2 Chip-Seq peaks or not in RPE. Such separation might allow pathway analysis for RPE-expressed genes and neural retina-expressed genes.

Minor points:

For the analysis, they claim to have used the Ensembl 93 software. Are they talking about the genome version? In that case, how did they analyzed the data? Which tools were used (EdgeR Deseq2 ... ?).

"The presence of versican, decorin, and biglycan in the list of P34 downregulated genes indicated that IPM was also affected” Based on the explanation provided before all genes used for pathways analysis were continuously downregulated from P30 to P38. Why talking about a list of P34 downregulated genes?

The authors should add gene symbols. It will be easier to find the expression profile of the corresponding genes in the heatmaps.

In results, Figure 2A described then 3B followed by 2C. It should be 2B, not 3B.

Fig 4 has only 3 panels (a, b, c) but the authors wrote legend for d and e.

In Mat and Meth:

The age when mice are injected should be added in the Animals part.

For RNA-Seq, can the authors mention the exact version of the genome used for mapping and analysis? The LogFC are corresponding to log2 or log10?

AAV vectors. “The soluble AP” they should introduce the abbreviation right at the beginning.

There is a typo mistake for VmdD2-SoAP. It should be Vmd2-SoAP. They should also indicate that the chosen promoter will drive OTX2 expression specifically in RPE.
